# A Novel MAGDM Method Based on Hesitant Picture Fuzzy Schweizer–Sklar Maclaurin Symmetric Mean Operators and Their Application

**DOI:** 10.3390/e24020238

**Published:** 2022-02-03

**Authors:** Tiedong Chen, Long Ye

**Affiliations:** School of Economics and Management, Beijing Jiaotong University, Beijing 100044, China; lye1@bjtu.edu.cn

**Keywords:** hesitant picture fuzzy set, Schweizer–Sklar *t*-norm and *t*-conorm, Maclaurin symmetric mean operators, MAGDM

## Abstract

Multiple attribute group decision making (MAGDM) issues play important roles in our daily life. In order to solve the problem that decision makers (DMs) may feel hesitant to select the appropriate evaluation values from several possible values in the process of providing evaluations, fuzzy theory and its extensions are widely applied in MAGDM problems. In this study, we first proposed hesitant picture fuzzy sets (HPFSs), which is a combination of the hesitant fuzzy set and picture fuzzy set. Subsequently, we introduced a novel Schweizer–Sklar *t*-norm and *t*-conorm operation rules of HPFSs and proposed a family of hesitant picture fuzzy Schweizer–Sklar Maclaurin symmetric mean operators. To show the application procedure of the proposed method to practical MAGDM issues, a numerical example about enterprise informatization level evaluation was employed to elaborate the calculation process with the proposed method. Finally, through the parameter analysis, validity analysis, and comparative analysis with some existing methods, we found that our method is more superior in providing DMs a greater decision-making freedom and relaxing the constraints on expressing personal preferences. This study provides a general framework of the proposed method to MAGDM problems under hesitant picture fuzzy environment, which enriches the fuzzy theory and its applications.

## 1. Introduction

Econophysics is a heterodox interdisciplinary research field, applying theories and methods originally developed by physicists in order to solve problems in economics, usually those including uncertainty or stochastic processes and nonlinear dynamics, which have received widespread attention from scholars [1,2,3]. In recent years, with the increasing complexity of the social economy, some scholars tend to apply fuzzy set theories into complex economic problems, and multiple-attribute decision making (MAGDM) is one of the hottest application areas. MAGDM is a situation faced when individuals collectively make a choice from the alternatives with respect to a set of attributes. MAGDM problems occupy important positions in the field of society decision making. For example, the investor wants to select the optimal company to avoid investment risk and obtain the maximum return, and the enterprise needs to evaluate its informatization level before enhancing it. Generally, all these activities need to be evaluated with respect to several attributes, which are also known as MAGDM problems and are very common in our daily life. Therefore, MAGDM problems have received great attentions in the past few decades [4,5,6]. With the increase in the complexity of the actual world, fuzzy set (FS) theory, proposed by Zadeh in 1965 [7], which was designed to utilize the membership degree (MD) to represent DMs’ expression information, has drawn more and more attention. FS theory provides a brand-new viewpoint for solving the internal uncertainty of experts’ cognition or insufficient information and the external ambiguity of coincidental chance events. From this foundation, the intuitionistic fuzzy set (IFS) which adds the no-membership degree (NMD) on the basis of FS was proposed by Atanassov [8], and the Pythagorean fuzzy set which relaxes the restrictions of IFS to the square sum of the MD and NMD cannot be larger than one was proposed by Yager [9]. These two fuzzy sets have also been applied in many fields since they were proposed [10,11]. Recently, Cuong proposed the picture fuzzy set (PFS) to fill in the gap of no neutral membership degree in IFS and Pythagorean fuzzy set [12]. The PFS further expands the freedom of decision makers (DMs) in the process of expressing their evaluation values, and the only constraint is that the sum of its three elements cannot be larger than one. Since its inception, some further studies according to PFS have been conducted. Singh proposed the correlation coefficients of PFS and tested the effectiveness in a bidirectional approximate reasoning system [13]. Son constructed a classification algorithm on the basis of PFS and applied it to the prediction problems [14]. Furthermore, Son proposed a generalized distance measure method of PFS and also applied it in clustering research [15]. Luo and Zhang designed a new similarity measure method of PFS and put into use in pattern-recognition experiments [16]. Ullah [17] applied the Maclaurin symmetric mean (MSM) operator to PFS environment and proposed a series of picture fuzzy MSM operators. Liu and Zhang [18] combined PFS with linguistic terms to describe the qualitative information. All the above studies provided the effectiveness of methods based on PFS.

In practical decision-making problems, DMs may feel hesitant to select the most appropriate one from several possible evaluation values. Although the theories introduced above make great contributions to the development of MAGDM problems, they can only include one value in every element, which may be insufficient for tackling DMs’ uncertainty cognition sometimes (e.g., one DM is hesitant to assign 0.2 or 0.3 as the evaluation value). To fill this gap, Torra proposed the hesitant fuzzy set (HFS) [19], which allows for full or partial admission when DMs vacillate among several valuations. As its NMD part is ignored, Peng et al. [20] combined the HFS with IFS and gave the definition of hesitant intuitionistic fuzzy set. Subsequently, Yang [21] et al. took another step forward and introduced the hesitant Pythagorean fuzzy set, as well as some of its aggregation operators. We have known that PFS is more powerful than IFS and the Pythagorean fuzzy set. Thus, the first question this paper aims to solve is how to combine the HFS and PFS and propose a method with a much wider decision-making space. Motivated by these, this paper plans to unite the HFS and the PFS and gives the concept of the hesitant picture fuzzy set (HPFS), which simultaneously adopts the advantages of HFS and PFS.

In addition to information expression, another key point of the MAGDM method is the information aggregation operator, which is used for concentrating on aggregating the evaluation values of alternatives with respect to the attributes. Therefore, the second question is how to establish a method to aggregate the evaluation information expressed by hesitant picture fuzzy elements. In the past decades, many related studies have been developed, such as the most traditional weighted average operator [22] and the geometric average operator [23], with the hypothesis that the input arguments are independent from each other, and the relative advanced Heronian mean operator [24] and the Bonferroni mean operator [25], which are skilled in considering interrelationships between any two attributes. The complexity of society deepens the individual’s cognition of the relationships among different things and gradually realizes that it may have an important impact on the final result. Under such circumstances, the Maclaurin symmetric mean (MSM) operator comes into the individual’s view due to its powerful relation processing capabilities [26,27]. Compared with the other operators, the priority of the MSM operator is that it can handle the number of attributes that have a correlation according to the actual situation with a flexible parameter variable. Thus, this paper decides to employ the MSM operator to aggregate the evaluation information expressed by HPFSs and propose a family of HPFS MSM operators. Moreover, the aggregation operators should be utilized with the operational laws. Thus, the third question is how to choose the appropriate operational rules with more flexibility. There are several kinds of operational rules, such as the simple algebraic rules, the Archimedean *t*-norm and *t*-conorm (ATT) rules [28], the Einstein *t*-norm and *t*-conorm rules [29], Dombi *t*-norm and *t*-conorm rules [30], Frank *t*-norm and *t*-conorm rules [31], Hamacher *t*-norm and *t*-conorm rules [32], etc. Moreover, the Schweizer–Sklar *t*-norm and *t*-conorm (SSTT) [33] operational rules are a special kind of ATT and are demonstrated to be very flexible and effective in dealing with fuzzy information with a parameter [34]. To our knowledge, it still has not been utilized in tackling the hesitant picture fuzzy information. Therefore, this paper proposes the operational rules of HPFSs and then a series of the HPF Schweizer–Sklar MSM (HPFSSMSM) operator, including the HPFSSMSM operator and its weighted form, and the dual form and the weighted dual form are defined accordingly.

The aims and motivations of this paper are: (1) to propose the concept of HPFS; (2) to propose some HPFSSMSM operators; (3) to propose an SSTT operation for HPF elements; and (4) to propose a novel method for MAGDM based on the proposed operators. Correspondingly, the contributions of this paper are: (1) we introduced a family of information aggregation operators by combining the HPFS and MSM operators based on SSTT operational rules; (2) we provided a general framework of the proposed method to MAGDM problems; and (3) we applied the proposed method to enterprise informatization level evaluation issues.

The rest of this paper is organized as follows: Section 2 recalls some basic notions and gives the SSTT operational rules based on HPFS. Section 3 proposes a battery of HPFSSMSM operators. Section 4 introduces a novel approach to MAGDM. Section 5 provides a numerical example to demonstrate the validity and merits of the proposed approach. Section 6 summarizes the paper.

## 2. Preliminaries

In this section, we recall the concepts of PFS, SSTT, and MSM operator, which are being utilized in the following section.

### 2.1. Picture Fuzzy Set and Hesitant Picture Fuzzy Set

**Definition** **1.** *Let X be an ordinary fixed set, a picture fuzzy set (PFS) A defined on X is given by* [12]

(1) $$A=\left\{\langle x,\mu_{A}\left(x\right),\eta_{A}\left(x\right),v_{A}\left(x\right)\rangle \;\left|x\in X\right.\right\},$$

*where* $\mu_{A}\left(x\right)$*,* $\eta_{A}\left(x\right)$ *and* $\;v_{A}\left(x\right)$ *represent the positive membership degree (PMD), neutral membership degree (NLMD) and negative membership degree (NEMD), respectively, satisfying* $\mu_{A}\left(x\right)\in \left[0,1\right]$, $\eta_{A}\left(x\right)\in \left[0,1\right]$, $v_{A}\left(x\right)\in \left[0,1\right]$ *and* $0\leq \mu_{A}\left(x\right)+\eta_{A}\left(x\right)+v_{A}\left(x\right)\leq 1$, ∀x∈X*. Then, for* x∈X, $\pi_{A}\left(x\right)=1-\left(\mu_{A}\left(x\right)+\eta_{A}\left(x\right)+v_{A}\left(x\right)\right)$ *is called the degree of refusal membership of x in A. For convenience,* $\alpha =\left(\mu_{\alpha },\eta_{\alpha },v_{\alpha }\right)$ *is called a picture fuzzy number (PFN), where* $\mu_{\alpha },\eta_{\alpha },v_{\alpha }\in \left[0,1\right]$ *and* $\mu_{\alpha }+\eta_{\alpha }+v_{\alpha }\leq 1$.

Motivated by Torra’s [19] HFS, we propose the concept of HPFS.

**Definition** **2.** *Let X be an ordinary fixed set, a hesitant picture fuzzy set (HPFS) B defined on X is defined as*

(2) $$B=\left\{\langle x,h_{B}\left(x\right),g_{B}\left(x\right),t_{B}\left(x\right)\rangle \;\left|x\in X\right.\right\},$$

*where*$h_{B}\left(x\right)$*,* $g_{B}\left(x\right)$ *and* $t_{B}\left(x\right)$ *are three sets of values, representing the PMD, NLMD, and NEMD of the element* x∈X *to the set B, respectively, satisfying* $\chi ,\phi ,\tau ,\in \left[0,1\right]$ *and*  maxχ+maxϕ+maxτ≤1*, where* $\chi \in h_{B}\left(x\right)$*,* $\phi \in g_{B}\left(x\right)$ *and* $\tau \in t_{B}\left(x\right)$*. For convenience, we call* $d=\left(h_{B}\left(x\right),g_{B}\left(x\right),t_{B}\left(x\right)\right)$ *a hesitant picture fuzzy elements (HPFE), which can be simply denoted as* $d=\left(h,g,t\right)$*, with the condition* χ∈h, ϕ∈g, τ∈t*,* $\chi ,\phi ,\tau ,\in \left[0,1\right]$ *and* maxχ+maxϕ+maxτ≤1

**Remark** **1.** *In particular, if* $\forall \phi \in g_{B}\left(x\right),\phi =0$*, then the HPFS reduces to the hesitant fuzzy set; if each of the collections* $h_{B}\left(x\right)$*,* $g_{B}\left(x\right)$ *and* $t_{B}\left(x\right)$ *contain only one element, then the HPFS reduces to the intuitionistic fuzzy set.*

In order to improve the readability, we provide the following example to illustrate the difference between PFS and HPFS.

**Example** **1.** *Suppose that in an MAGDM problem, one DM directly provides his/her evaluation with a PFN* $\alpha_{1}=\left(0.2,0.3,0.4\right)$*, but another DM feels uncertain among whether to provide 0.2 or 0.3 as the membership degree. In this situation, we find that the PFN is insufficient to express the real opinion of the second DM. Thus, we can employ the proposed HPFE* $d_{2}=\left\{\left\{0.2,0.3\right\},\left\{0.3\right\},\left\{0.4\right\}\right\}$ *as the assessment. On the other hand, we can also utilize* $d_{1}=\left\{\left\{0.2,0.2\right\},\left\{0.3\right\},\left\{0.4\right\}\right\}$ *to express the evaluation value of the first DM, because the essence of* $d_{1}$ *and* $\alpha_{1}$ *is the same, which is consistent with Remark 1. Therefore, the proposed HPFS is more flexible than PFS*.

To compare any two HPFEs, we provide a comparison law for HPFEs.

**Definition** **3.** *Let* $d=\left(h,g,t\right)$ *be an HPFE, then* $S\left(d\right)=\frac{1}{\#h}\sum_{\chi \in h}\chi -\frac{1}{\#t}\sum_{\tau \in t}\phi$ *is the score function of d and* $H\left(d\right)=\frac{1}{\#h}\sum_{\chi \in h}\chi +\frac{1}{\#g}\sum_{\phi \in g}\phi +\frac{1}{\#t}\sum_{\tau \in t}\tau$ *is the accuracy function of d respectively, where* #h*, #g, and #t denote the numbers of values in h, g, and t, respectively. Let* $d_{1}=\left(h_{1},g_{1},t_{1}\right)$ *and* $d_{2}=\left(h_{2},g_{2},t_{2}\right)$ *be any two HPFEs,* $S\left(d_{1}\right)$ *and* $S\left(d_{2}\right)$ *be score functions of d_1_ and d_2_, respectively,* $H\left(d_{1}\right)$ *and* $H\left(d_{2}\right)$*be accuracy function of d_1_ and d_2_, respectively. Then,*

1. *If* $S\left(d_{1}\right)>S\left(d_{2}\right)$*, then* $d_{1}>d_{2}$*;*
2. *If* $S\left(d_{1}\right)>S\left(d_{2}\right)$*,* *then*
  *if* $H\left(d_{1}\right)>H\left(d_{2}\right)$*, then* $d_{1}>d_{2}$*;*
  *if* $H\left(d_{1}\right)=H\left(d_{2}\right)$*, then* $d_{1}=d_{2}$

In the following, we provide an example to show the calculation details of Definition 3.

**Example** **2.** *Suppose that there are two HPFEs* $d_{1}=\left\{\left\{0.2,0.3\right\},\left\{0.3\right\},\left\{0.4\right\}\right\}$ *and* $d_{2}=\left\{\left\{0.4,0.5\right\},\left\{0.3\right\},\left\{0.1,0.2\right\}\right\}$*, then we can obtain that*

$$S\left(d_{1}\right)=\frac{1}{2}\times \left(0.2+0.3\right)-0.4=-0.15\;\mathrm{and}\;S\left(d_{2}\right)=\frac{1}{2}\times \left(0.4+0.5\right)-\frac{1}{2}\times \left(0.1+0.2\right)=0.3$$

*Then, we can obtain* $S\left(d_{1}\right)<S\left(d_{2}\right)$*, so that* $d_{1}<d_{2}$.

### 2.2. Schweizer–Sklar t-Norm and t-Conorm

The definitions of Schweizer–Sklar *t*-norm and *t*-conorm (SSTT) are presented as follows:

(3) $$T_{ss,\gamma }\left(x,y\right)={\left(x^{\gamma }+y^{\gamma }-1\right)}^{1/\gamma };$$

(4) $$T_{ss,\gamma }^{*}\left(x,y\right)=1-{\left({\left(1-x\right)}^{\gamma }+{\left(1-y\right)}^{\gamma }-1\right)}^{1/\gamma };$$

where γ<0, $x,y\in \left[0,1\right]$. Additionally, when γ→0, we have $T_{\gamma }\left(x,y\right)=xy$ and $T_{\gamma }^{*}\left(x,y\right)=x+y-xy$. They are the algebraic *t*-norm and algebraic *t*-conorm (ATT).

Based on the Schweizer–Sklar *t*-norm and *t*-conorm, we provide some Schweizer–Sklar operations for HPFEs.

**Definition** **4.** *Let*$d=\left(h,g,t\right)$*,* $d_{1}=\left(h_{1},g_{1},t_{1}\right)$ *and* $d_{2}=\left(h_{2},g_{2},t_{2}\right)$ *be any three HPFEs,* γ *be a negative real number and n be positive real number, then*

1. $d_{1}⊕d_{2}=\cup_{\chi_{1}\in h_{1},\chi_{2}\in h_{2},\phi_{1}\in g_{1},\phi_{2}\in g_{2},\tau_{1}\in t_{1},\tau_{2}\in t_{2}}$$\left\{\left\{1-{\left({\left(1-\chi_{1}\right)}^{\gamma }+{\left(1-\chi_{2}\right)}^{\gamma }-1\right)}^{1/\gamma }\right\},\left\{{\left(\phi_{1}{}^{\gamma }+\phi_{2}{}^{\gamma }-1\right)}^{1/\gamma }\right\},\left\{{\left(\tau_{1}{}^{\gamma }+\tau_{2}{}^{\gamma }-1\right)}^{1/\gamma }\right\}\right\};$
2. $d_{1}⊗d_{2}=\cup_{\chi_{1}\in h_{1},\chi_{2}\in h_{2},\phi_{1}\in g_{1},\phi_{2}\in g_{2},\tau_{1}\in t_{1},\tau_{2}\in t_{2}}$$\left\{\left\{{\left(\chi_{1}{}^{\gamma }+\chi_{2}{}^{\gamma }-1\right)}^{1/\gamma }\right\},\left\{1-{\left({\left(1-\phi_{1}\right)}^{\gamma }+{\left(1-\phi_{2}\right)}^{\gamma }-1\right)}^{1/\gamma }\right\},\left\{1-{\left({\left(1-\tau_{1}\right)}^{\gamma }+{\left(1-\tau_{2}\right)}^{\gamma }-1\right)}^{1/\gamma }\right\}\right\};$
3. $nd=\cup_{\chi \in h,\phi \in g,\tau \in t}\left\{\left\{1-{\left(n{\left(1-\chi \right)}^{\gamma }-\left(n-1\right)\right)}^{1/\gamma }\right\},\left\{{\left(n\phi^{\gamma }-\left(n-1\right)\right)}^{1/\gamma }\right\},\left\{{\left(n\tau^{\gamma }-\left(n-1\right)\right)}^{1/\gamma }\right\}\right\};$
4. $d^{n}=\cup_{\chi \in h,\phi \in g,\tau \in t}\left\{\left\{{\left(n\chi^{\gamma }-\left(n-1\right)\right)}^{1/\gamma }\right\},\left\{1-{\left(n{\left(1-\phi \right)}^{\gamma }-\left(n-1\right)\right)}^{1/\gamma }\right\},\left\{1-{\left(n{\left(1-\tau \right)}^{\gamma }-\left(n-1\right)\right)}^{1/\gamma }\right\}\right\}$

**Example** **3.** *Suppose that there are two HPFE* $d_{1}=\left\{\left\{0.2,0.3\right\},\left\{0.3\right\},\left\{0.4\right\}\right\}$ *and* $d_{2}=\left\{\left\{0.4,0.5\right\},\left\{0.3\right\},\left\{0.1,0.2\right\}\right\}$*. Let* γ=−1 *and* n=2 *; then, we can obtain that*

$$d_{1}⊕d_{2}=\left\{\begin{array}{l} \left\{\begin{array}{l} 1-{\left({\left(1-0.2\right)}^{-1}+{\left(1-0.4\right)}^{-1}-1\right)}^{-1},1-{\left({\left(1-0.2\right)}^{-1}+{\left(1-0.5\right)}^{-1}-1\right)}^{-1},\\ 1-{\left({\left(1-0.3\right)}^{-1}+{\left(1-0.4\right)}^{-1}-1\right)}^{-1},1-{\left({\left(1-0.3\right)}^{-1}+{\left(1-0.5\right)}^{-1}-1\right)}^{-1} \end{array}\right\},\\ \left\{{\left(0.3^{-1}+0.3^{-1}-1\right)}^{-1}\right\},\left\{{\left(0.4^{-1}+0.1^{-1}-1\right)}^{-1},{\left(0.4^{-1}+0.2^{-1}-1\right)}^{-1}\right\} \end{array}\right\}\;=\left\{\left\{0.4783,0.5556,0.5227,0.5882\right\},\left\{0.1765\right\},\left\{0.0870,0.1538\right\}\right\},$$

*Similarly, we can obtain that*

$$d_{1}⊗d_{2}=\left\{\left\{0.1538,0.1667,0.2069,0.2308\right\},\left\{0.4615\right\},\left\{0.4375,0.4783\right\}\right\},$$

$$nd_{1}=\left\{\left\{0.3333,0.4615\right\},\left\{0.1765\right\},\left\{0.2500\right\}\right\},$$

$$d_{1}^{n}=\left\{\left\{0.1111,0.1765\right\},\left\{0.4615\right\},\left\{0.5714\right\}\right\}.$$

According to Definition 4, the following theorem can be derived.

**Theorem** **1.** *Let*$d=\left(h,g,t\right)$*,* $d_{1}=\left(h_{1},g_{1},t_{1}\right)$ *and* $d_{2}=\left(h_{2},g_{2},t_{2}\right)$ *be any three HPFEs,* $\gamma_{1}$ *and* $\gamma_{2}$ *be two positive real numbers, then*

1. $d_{1}⊕d_{2}=d_{2}⊕d_{1}$;
2. $d_{1}⊗d_{2}=d_{2}⊗d_{1}$;
3. $\gamma \left(d_{1}⊕d_{2}\right)=\gamma d_{1}⊕\gamma d_{2},\gamma >0$;
4. $\gamma_{1}d⊕\gamma_{2}d=\left(\gamma_{1}⊕\gamma_{2}\right)d,\gamma_{1}>0,\gamma_{2}>0$;
5. $d^{\gamma_{1}}⊗d^{\gamma_{2}}=d^{\gamma_{1}+\gamma_{2}},\gamma_{1}>0,\gamma_{2}>0$;
6. $d_{1}^{\gamma }⊗d_{2}^{\gamma }={\left(d_{1}⊗d_{2}\right)}^{\gamma },\gamma >0$;

### 2.3. Maclaurin Symmetric Mean

The MSM was firstly proposed by Maclaurin [27] for crisp numbers. The prominent feature of the MSM is that it can capture the interrelationship among the aggregated arguments.

**Definition** **5.** [27] *Let* $a_{j}\left(j=1,2,\ldots ,n\right)$ *be a collection of crisp numbers, and* k=1,2,…,n*. If*

(5) $$MSM^{\left(k\right)}\left(a_{1},a_{2},\ldots ,a_{n}\right)={\left(\frac{\sum_{1\leq i_{1}<\ldots <i_{k}\leq n}\prod_{j=1}^{k}a_{i_{j}}}{C_{n}^{k}}\right)}^{1/k},$$

*then* $MSM^{\left(k\right)}$ *is called the MSM, where* $\left(i_{1},i_{2},\ldots ,i_{k}\right)$ *traversal all the k-tuple combination of (1, 2, …, n),* $C_{n}^{k}$ *is the binomial coefficient*.

In addition, Qin and Liu [35] proposed the dual Maclaurin symmetric mean.

**Definition** **6.** *Let* $a_{j}\left(j=1,2,\ldots ,n\right)$ *be a collection of crisp numbers, and* k=1,2,…,n. [35] *If*

(6) $$DMSM^{\left(k\right)}\left(a_{1},a_{2},\ldots ,a_{n}\right)=\frac{1}{k}{\left(\prod_{1\leq i_{1}<\ldots <i_{k}\leq n}\sum_{j=1}^{k}a_{i_{j}}\right)}^{1/C_{n}^{k}},$$

*then,* $DMSM^{\left(k\right)}$ *is called DMSM, where* $\left(i_{1},i_{2},\ldots ,i_{k}\right)$ *traversal all the k-tuple combination of (1, 2, …, n),* $C_{n}^{k}$ *is the binomial coefficient.*

## 3. The Hesitant Picture Fuzzy Schweizer–Sklar Maclaurin Symmetric Mean Operators

In this section, we extend the MSM to hesitant picture fuzzy environment and propose a family of hesitant picture fuzzy Maclaurin symmetric mean operators. Moreover, some desirable properties and special cases of the proposed aggregation operators are investigated.

### 3.1. The Hesitant Picture Fuzzy Schweizer–Sklar Maclaurin Symmetric Mean (HPFSSMSM) Operator

**Definition** **7.** *Let* $d_{j}=\left(h_{j},g_{j},t_{j}\right)$ (j=1,2,…,n) *be a collection of HPFEs, and* k=1,2,…,n*, then the operator is defined as*

(7) $$HPFSSMSM^{\left(k\right)}\left(d_{1},d_{2},\ldots ,d_{n}\right)={\left(\frac{\sum_{1\leq i_{1}<\ldots <i_{k}\leq n}\prod_{j=1}^{k}d_{i_{j}}}{C_{n}^{k}}\right)}^{1/k},$$

*where* $\left(i_{1},i_{2},\ldots ,i_{k}\right)$ *traversal all the k-tuple combination of* $\left(1,2,\ldots ,n\right)$ *and* $C_{n}^{k}$ *is the binomial coefficient.*

Based on the operations for HPFEs, the following theorem can be obtained.

**Theorem** **2.** *Let* $d_{j}=\left(h_{j},g_{j},t_{j}\right)$ (j=1,2,…,n) *be a collection of HPFEs, and* k=1,2,…,n*, then the aggregated value by the HPFSSMSM operator is still an HPFE and*

(8) $$HPFSSMSM^{\left(k\right)}\left(d_{1},d_{2},\ldots ,d_{n}\right)=\;\cup_{\chi_{i_{j}}\in h_{i_{j}},\phi_{i_{j}}\in g_{i_{j}},\tau_{i_{j}}\in t_{i_{j}}}\left\{\left\{{\left(\frac{1}{k}{\left(1-{\left(\frac{1}{C_{n}^{k}}\sum_{1\leq i_{1}<\ldots <i_{k}\leq n}{\left(1-{\left(\sum_{j=1}^{k}\chi_{i_{j}}-1\right)}^{\frac{1}{\gamma }}\right)}^{\gamma }+1\right)}^{\frac{1}{\gamma }}\right)}^{\gamma }-\left(\frac{1}{k}-1\right)\right)}^{\frac{1}{\gamma }}\right\},\right.\;\left\{1-{\left(\frac{1}{k}{\left(1-{\left(\frac{1}{C_{n}^{k}}\sum_{1\leq i_{1}<\ldots <i_{k}\leq n}{\left(1-{\left(\sum_{j=1}^{k}{\left(1-\phi_{i_{j}}\right)}^{\gamma }-1\right)}^{\frac{1}{\gamma }}\right)}^{\gamma }+1\right)}^{\frac{1}{\gamma }}\right)}^{\gamma }-\left(\frac{1}{k}-1\right)\right)}^{\frac{1}{\gamma }}\right\},\;\left.\left\{1-{\left(\frac{1}{k}{\left(1-{\left(\frac{1}{C_{n}^{k}}\sum_{1\leq i_{1}<\ldots <i_{k}\leq n}{\left(1-{\left(\sum_{j=1}^{k}{\left(1-\tau_{i_{j}}\right)}^{\gamma }-1\right)}^{\frac{1}{\gamma }}\right)}^{\gamma }+1\right)}^{\frac{1}{\gamma }}\right)}^{\gamma }-\left(\frac{1}{k}-1\right)\right)}^{\frac{1}{\gamma }}\right\}\right\}.$$

The detailed proof can be found in Appendix A (Proof of Theorem 2).

In the following, we discuss some desirable properties of the HPFSSMSM operator.

**Theorem** **3.** *(Idempotency). Let*$d_{j}=d=\left(h,g,t\right)$, *for all*j=1,2,…,n*, then*

(9) $$HPFSSMSM^{\left(k\right)}\left(d_{1},d_{2},\ldots ,d_{n}\right)=d$$

The detailed proof can be found in Appendix A (Proof of Theorem 3).

**Theorem** **4.** *(Monotonicity). Let* $d_{j}=\left(h_{j},g_{j},t_{j}\right)$ *and* $d_{j}^{\prime }=\left(h_{j}^{\prime },g_{j}^{\prime },t_{j}^{\prime }\right)$ *be two sets of HPFEs, if* $d_{j}\geq d_{j}^{\prime }$ *holds for all* j=1,2,…,n*, then*

(10) $$HPFSSMSM^{\left(k\right)}\left(d_{1},d_{2},\ldots ,d_{n}\right)\geq HPFSSMSM^{\left(k\right)}\left(d_{1}^{\prime },d_{2}^{\prime },\ldots ,d_{n}^{\prime }\right).$$

The detailed proof can be found in Appendix A (Proof of Theorem 4).

**Theorem** **5.** *(Boundedness). Let* $d_{j}=\left(h_{j},g_{j},t_{j}\right)$ (j=1,2,…,n) *be a collection of HPFEs, if* $d^{-}=\underset{j}{\min}d_{j}$ *and* $d^{+}=\underset{j}{\max}d_{j}$ *then*

(11) $$d^{-}\leq HPFSSMSM^{\left(k\right)}\left(d_{1},d_{2},\ldots ,d_{n}\right)\leq d^{+}.$$

The detailed proof can be found in Appendix A (Proof of Theorem 5).

In the following section, we shall discuss some special cases of the HPFSSMSM operator based on the different values of the parameter *k*.

**Case** **1.** If γ→0, the HPFSSMSM is reduced to the hesitant picture fuzzy MSM (HPFMSM) operator.
  (12) $$HPFSSMSM^{\left(k\right)}\left(d_{1},d_{2},\ldots ,d_{n}\right)=\;=\cup_{\chi_{i_{j}}\in h_{i_{j}},\phi_{i_{j}}\in g_{i_{j}},\tau_{i_{j}}\in t_{i_{j}}}\left\{\left\{{\left(1-{\prod_{1\leq i_{1}<\ldots <i_{k}\leq n}^{}\left(1-\prod_{j=1}^{k}\chi_{ij}\right)}^{\frac{1}{C_{n}^{k}}}\right)}^{\frac{1}{k}}\right\},\;\left\{1-{\left(1-{\prod_{1\leq i_{1}<\ldots <i_{k}\leq n}^{}\left(1-\prod_{j=1}^{k}\left(1-\phi_{ij}\right)\right)}^{\frac{1}{C_{n}^{k}}}\right)}^{\frac{1}{k}}\right\},\right.\left.\left\{1-{\left(1-{\prod_{1\leq i_{1}<\ldots <i_{k}\leq n}^{}\left(1-\prod_{j=1}^{k}\left(1-\tau_{ij}\right)\right)}^{\frac{1}{C_{n}^{k}}}\right)}^{\frac{1}{k}}\right\}\right\}.$$
**Case** **2.** If *k* = 1, based on the definition of HPFSSMSM, we have
  (13) $$HPFSSMSM^{\left(1\right)}\left(d_{1},d_{2},\ldots ,d_{n}\right)=\frac{1}{n}\sum_{i=1}^{n}d_{i}=\cup_{\chi_{i_{j}}\in h_{i_{j}},\phi_{i_{j}}\in g_{i_{j}},\tau_{i_{j}}\in t_{i_{j}}}\left\{\left\{1-{\left(\frac{1}{n}\sum_{i=1}^{n}{\left(1-\chi_{i}\right)}^{\gamma }-\frac{2}{n}+1\right)}^{\frac{1}{\gamma }}\right\},\right.\;\left.\left\{{\left(\frac{1}{n}\sum_{i=1}^{n}\phi_{i_{j}}^{\gamma }-\frac{2}{n}+1\right)}^{\frac{1}{\gamma }}\right\},\left\{{\left(\frac{1}{n}\sum_{i=1}^{n}\tau_{i_{j}}^{\gamma }-\frac{2}{n}+1\right)}^{\frac{1}{\gamma }}\right\}\right\}.$$
  In this case, the HPFSSMSM reduces to the hesitant picture fuzzy Schweizer–Sklar averaging (HPFSSA) operator. If γ→0, the HPFSSMSM operator is reduced to hesitant picture fuzzy averaging operator.
  (14) $$HPFSSMSM^{\left(1\right)}\left(d_{1},d_{2},\ldots ,d_{n}\right)=\frac{1}{n}\sum_{i=1}^{n}d_{i}=\cup_{\chi_{i_{j}}\in h_{i_{j}},\phi_{i_{j}}\in g_{i_{j}},\tau_{i_{j}}\in t_{i_{j}}}\;\left\{\left\{1-{\left(\prod_{i=1}^{n}\left(1-\chi_{i}\right)\right)}^{\frac{1}{n}}\right\},\left\{{\left(\prod_{i=1}^{n}\phi_{i}\right)}^{\frac{1}{n}}\right\},\left\{{\left(\prod_{i=1}^{n}\tau_{i}\right)}^{\frac{1}{n}}\right\}\right\}.$$
**Case** **3.** If *k* = 2, based on the definition of HPFSSMSM, we have
  (15) $$HPFSSMSM^{\left(2\right)}\left(d_{1},d_{2},\ldots ,d_{n}\right)={\left(\frac{1}{n(n-1)}\sum_{\begin{array}{l} i_{1},i_{2}=1\\ i_{1}\neq i_{2} \end{array}}^{n}\left(d_{i_{1}}d_{i_{2}}\right)\right)}^{\frac{1}{2}}=\cup_{\chi_{i_{1}}\in h_{i_{1}},\chi_{i_{2}}\in h_{i_{2}},\phi_{i_{1}}\in g_{i_{1}},\phi_{i_{2}}\in g_{i_{2}},\tau_{i_{1}}\in t_{i_{1}},\tau_{i_{2}}\in t_{i_{2}}}\;\left\{\left\{{\left(\frac{1}{2}{\left(1-{\left(\frac{1}{n(n-1)}\sum_{\begin{array}{l} i_{1},i_{2}=1\\ i_{1}\neq i_{2} \end{array}}^{n}{\left(1-{\left(\chi_{i_{1}}^{\gamma }+\chi_{i_{2}}^{\gamma }-1\right)}^{\frac{1}{\gamma }}\right)}^{\gamma }+1\right)}^{\frac{1}{\gamma }}\right)}^{\gamma }+\frac{1}{2}\right)}^{\frac{1}{\gamma }}\right\},\right.\;\left\{1-{\left(\frac{1}{2}{\left(1-{\left(\frac{1}{n(n-1)}\sum_{\begin{array}{l} i_{1},i_{2}=1\\ i_{1}\neq i_{2} \end{array}}^{n}{\left(1-{\left({\left(1-\phi_{i_{1}}\right)}^{\gamma }+{\left(1-\phi_{i_{2}}\right)}^{\gamma }-1\right)}^{\frac{1}{\gamma }}\right)}^{\gamma }+1\right)}^{\frac{1}{\gamma }}\right)}^{\gamma }+\frac{1}{2}\right)}^{\frac{1}{\gamma }}\right\},\;\left.\left\{1-{\left(\frac{1}{2}{\left(1-{\left(\frac{1}{n(n-1)}\sum_{\begin{array}{l} i_{1},i_{2}=1\\ i_{1}\neq i_{2} \end{array}}^{n}{\left(1-{\left({\left(1-\tau_{i_{1}}\right)}^{\gamma }+{\left(1-\tau_{i_{2}}\right)}^{\gamma }-1\right)}^{\frac{1}{\gamma }}\right)}^{\gamma }+1\right)}^{\frac{1}{\gamma }}\right)}^{\gamma }+\frac{1}{2}\right)}^{\frac{1}{\gamma }}\right\}\right\}.$$
  In this case, the HPFSSMSM reduces to the hesitant picture fuzzy Schweizer–Sklar Bonferroni mean (HPFSSBM) operator. If γ→0, the HPFSSMSM operator is reduced to the hesitant picture fuzzy Bonferroni mean operator.
  (16) $$HPFSSMSM^{\left(2\right)}\left(d_{1},d_{2},\ldots ,d_{n}\right)={\left(\frac{1}{n(n-1)}\sum_{\begin{array}{l} i_{1},i_{2}=1\\ i_{1}\neq i_{2} \end{array}}^{n}\left(d_{i_{1}}d_{i_{2}}\right)\right)}^{\frac{1}{2}}=\cup_{\chi_{i_{1}}\in h_{i_{1}},\chi_{i_{2}}\in h_{i_{2}},\phi_{i_{1}}\in g_{i_{1}},\phi_{i_{2}}\in g_{i_{2}},\tau_{i_{1}}\in t_{i_{1}},\tau_{i_{2}}\in t_{i_{2}}}\;\left\{\left\{{\left(1-{\prod_{\begin{array}{l} i_{1},i_{2}=1\\ i_{1}\neq i_{2} \end{array}}^{n}\left(1-\chi_{i_{1}}\chi_{i_{2}}\right)}^{\frac{1}{n(n-1)}}\right)}^{\frac{1}{2}}\right\},\left\{1-{\left(1-{\prod_{\begin{array}{l} i_{1},i_{2}=1\\ i_{1}\neq i_{2} \end{array}}^{n}\left(1-\left(1-\phi_{i_{1}}\right)\left(1-\phi_{i_{2}}\right)\right)}^{\frac{1}{n(n-1)}}\right)}^{\frac{1}{2}}\right\},\right.\;\left.\left\{1-{\left(1-{\prod_{\begin{array}{l} i_{1},i_{2}=1\\ i_{1}\neq i_{2} \end{array}}^{n}\left(1-\left(1-\tau_{i_{1}}\right)\left(1-\tau_{i_{2}}\right)\right)}^{\frac{1}{n(n-1)}}\right)}^{\frac{1}{2}}\right\}\right\}.$$
**Case** **4.** If *k* = 3, based on the definition of HPFSSMSM, we have
  (17) $$HPFSSMSM^{\left(3\right)}\left(d_{1},d_{2},\ldots ,d_{n}\right)={\left(\sum_{i_{1},i_{2},i_{3}=1}^{n}\left(d_{i_{1}}d_{i_{2}}d_{i_{3}}\right)\right)}^{\frac{1}{3}}=\cup_{\chi_{i_{1}}\in h_{i_{1}},\chi_{i_{2}}\in h_{i_{2}},\chi_{i_{3}}\in h_{i_{3}},\phi_{i_{1}}\in g_{i_{1}},\phi_{i_{2}}\in g_{i_{2}},\phi_{i_{3}}\in g_{i_{3}},,\tau_{i_{1}}\in t_{i_{1}},\tau_{i_{2}}\in t_{i_{2}},\tau_{i_{3}}\in t_{i_{3}}}\;\left\{\left\{{\left(\frac{1}{3}{\left(1-{\left(\sum_{i_{1},i_{2},i_{3}=1}^{n}{\left(1-{\left(\chi_{i_{1}}^{\gamma }+\chi_{i_{2}}^{\gamma }+\chi_{i_{3}}^{\gamma }-1\right)}^{\frac{1}{\gamma }}\right)}^{\gamma }-1\right)}^{\frac{1}{\gamma }}\right)}^{\gamma }+\frac{2}{3}\right)}^{\frac{1}{\gamma }}\right\},\right.\;\left\{1-{\left(\frac{1}{3}{\left(1-{\left(\sum_{i_{1},i_{2},i_{3}=1}^{n}{\left(1-{\left({\left(1-\phi_{i_{1}}\right)}^{\gamma }+{\left(1-\phi_{i_{2}}\right)}^{\gamma }+{\left(1-\phi_{i_{3}}\right)}^{\gamma }-1\right)}^{\frac{1}{\gamma }}\right)}^{\gamma }-1\right)}^{\frac{1}{\gamma }}\right)}^{\gamma }+\frac{2}{3}\right)}^{\frac{1}{\gamma }}\right\},\;\left.\left\{1-{\left(\frac{1}{3}{\left(1-{\left(\sum_{i_{1},i_{2},i_{3}=1}^{n}{\left(1-{\left({\left(1-\tau_{i_{1}}\right)}^{\gamma }+{\left(1-\tau_{i_{2}}\right)}^{\gamma }+{\left(1-\tau_{i_{3}}\right)}^{\gamma }-1\right)}^{\frac{1}{\gamma }}\right)}^{\gamma }-1\right)}^{\frac{1}{\gamma }}\right)}^{\gamma }+\frac{2}{3}\right)}^{\frac{1}{\gamma }}\right\}\right\}.$$
  In this case, the HFPSSMSM reduces to the hesitant picture fuzzy Schweizer–Sklar generalized Bonferroni mean (HFPSSGBM) operator. If γ→0, the HPFSSMSM operator is reduced to hesitant picture fuzzy generalized Bonferroni mean operator.
  (18) $$HPFSSMSM^{\left(3\right)}\left(d_{1},d_{2},\ldots ,d_{n}\right)={\left(\sum_{i_{1},i_{2},i_{3}=1}^{n}\left(d_{i_{1}}d_{i_{2}}d_{i_{3}}\right)\right)}^{\frac{1}{3}}=\cup_{\chi_{i_{1}}\in h_{i_{1}},\chi_{i_{2}}\in h_{i_{2}},\chi_{i_{3}}\in h_{i_{3}},\phi_{i_{1}}\in g_{i_{1}},\phi_{i_{2}}\in g_{i_{2}},\phi_{i_{3}}\in g_{i_{3}},,\tau_{i_{1}}\in t_{i_{1}},\tau_{i_{2}}\in t_{i_{2}},\tau_{i_{3}}\in t_{i_{3}}}\;\left\{\left\{{\left(1-\prod_{i_{1},i_{2},i_{3}=1}^{n}\left(1-\chi_{i_{1}}\chi_{i_{2}}\chi_{i_{3}}\right)\right)}^{\frac{1}{3}}\right\},\left\{1-{\left(1-\prod_{i_{1},i_{2},i_{3}=1}^{n}\left(1-\left(1-\phi_{i_{1}}\right)\left(1-\phi_{i_{2}}\right)\left(1-\phi_{i_{3}}\right)\right)\right)}^{\frac{1}{3}}\right\},\right.\;\left.\left\{1-{\left(1-\prod_{i_{1},i_{2},i_{3}=1}^{n}\left(1-\left(1-\tau_{i_{1}}\right)\left(1-\tau_{i_{2}}\right)\left(1-\tau_{i_{3}}\right)\right)\right)}^{\frac{1}{3}}\right\}\right\}.$$
**Case** **5.** If *k* = *n*, based on the definition of HPFSSMSM, we have
  (19) $$HPFSSMSM^{\left(n\right)}\left(d_{1},d_{2},\ldots ,d_{n}\right)={\left(\prod_{i=1}^{n}d_{i}\right)}^{\frac{1}{n}}=\cup_{\chi_{i}\in h_{i},\phi_{i}\in g_{i},\tau_{i}\in t_{i}}\left\{\left\{{\left(\frac{1}{n}\sum_{i=1}^{n}\chi_{i}^{\gamma }-\frac{2}{n}+1\right)}^{\frac{1}{\gamma }}\right\},\right.\;\left.\left\{1-{\left(\frac{1}{n}\sum_{i=1}^{n}{\left(1-\phi_{i}\right)}^{\gamma }-\frac{2}{n}+1\right)}^{\frac{1}{\gamma }}\right\},\left\{1-{\left(\frac{1}{n}\sum_{i=1}^{n}{\left(1-\tau_{i}\right)}^{\gamma }-\frac{2}{n}+1\right)}^{\frac{1}{\gamma }}\right\}\right\}.$$
  In this case, the HPFSSMSM reduces to the hesitant picture fuzzy Schweizer–Sklar geometric (HPFSSG) operator. If γ→0, the HPFSSMSM operator is reduced to the hesitant picture fuzzy geometric operator.
  (20) $$HPFSSMSM^{\left(n\right)}\left(d_{1},d_{2},\ldots ,d_{n}\right)={\left(\prod_{i=1}^{n}d_{i}\right)}^{\frac{1}{n}}=\cup_{\chi_{i}\in h_{i},\phi_{i}\in g_{i},\tau_{i}\in t_{i}}\;\left\{\left\{{\left(\prod_{i=1}^{n}\chi_{i}\right)}^{\frac{1}{n}}\right\},\left\{1-{\left(\prod_{i=1}^{n}\left(1-\phi_{i}\right)\right)}^{\frac{1}{n}}\right\},\left\{1-{\left(\prod_{i=1}^{n}\left(1-\tau_{i}\right)\right)}^{\frac{1}{n}}\right\}\right\}.$$
  From the above analysis, we can further discuss the monotonicity of the HPFSSMSM operator with respect to the parameter *k*. First, we introduce two lemmas which are used in the following discussion.

**Lemma** **1.** *Let*$a_{j}>0$*,* $b_{j}>0\left(j=1,2,\ldots ,n\right)$*, and* $\sum_{j=1}^{n}b_{j}=1$*, then* [36]

(21) $$\prod_{j=1}^{n}a_{j}^{b_{j}}\leq \sum_{j=1}^{n}a_{j}b_{j},$$

*with equality if and only if* $a_{1}=a_{2}=\ldots =a_{n}$.

**Theorem** **6.** *For the given HPFEs* $d_{j}\left(j=1,2,\ldots ,n\right)$ *and* k=1,2,…,n*, the HPFSSMSM is monotonically decreasing function with the increase in the parameter k.*

The detailed proof can be found in Appendix A (Proof of Theorem 6).

### 3.2. The Hesitant Picture Fuzzy Schweizer–Sklar-Weighted Maclaurin Symmetric Mean (HPFSSWMSM) Operator

The advantage of the HPFSSMSM operator is that it can decide the interrelationship among how many arguments can be considered with a parameter *k*. However, the HPFSSMSM operator does not consider the self-importance of the aggregated arguments. Therefore, we introduce the HFPSSWMSM operator, which can take the corresponding weights of aggregated HPFS into consideration.

**Definition** **8.** *Let*$d_{j}=\left(h_{j},g_{j},t_{j}\right)$(j=1,2,…,n)*be a collection of HPFEs, and* k=1,2,…,n. $w={\left(w_{1},w_{2},\ldots ,w_{n}\right)}^{T}$ *be the weight vector of* $d_{j}$*, satisfying* $w_{i}\in \left[0,1\right]$ *and* $\sum_{i=1}^{n}w_{i}=1$*, then the operator is defined as*

(22) $$HPFSSWMSM^{\left(k\right)}\left(d_{1},d_{2},\ldots ,d_{n}\right)={\left(\frac{\sum_{1\leq i_{1}<\ldots <i_{k}\leq n}\prod_{j=1}^{k}w_{i_{j}}d_{i_{j}}}{C_{n}^{k}}\right)}^{1/k}.$$

*where* $\left(i_{1},i_{2},\ldots ,i_{k}\right)$ *traversal all the k-tuple combination of* $\left(1,2,\ldots ,n\right)$ *and* $C_{n}^{k}$ *is the binomial coefficient.*

According to the operations of HPFEs, the aggregated value by the HPFSSWMSM can be obtained, which is shown as Theorem 7.

**Theorem** **7.** *Let* $d_{j}=\left(h_{j},g_{j},t_{j}\right)$ (j=1,2,…,n) *be a collection of HPFEs, and* k=1,2,…,n*, then the aggregated value by the HPFSSWMSM operator is still an HPFE and*

(23) $$HPFSSWMSM^{\left(k\right)}\left(d_{1},d_{2},\ldots ,d_{n}\right)=\cup_{\chi_{i_{j}}\in h_{i_{j}},\phi_{i_{j}}\in g_{i_{j}},\tau_{i_{j}}\in t_{i_{j}}}.\;\left\{\left\{{\left(\frac{1}{k}{\left(1-{\left(\frac{1}{C_{n}^{k}}\sum_{1\leq i_{1}<\ldots <i_{k}\leq n}{\left(1-{\left(\sum_{j=1}^{k}{\left(1-{\left(w_{i_{j}}{\left(1-\chi_{i_{j}}\right)}^{\gamma }-\left(w_{i_{j}}-1\right)\right)}^{\frac{1}{\gamma }}\right)}^{\gamma }-1\right)}^{\frac{1}{\gamma }}\right)}^{\gamma }+1\right)}^{\frac{1}{\gamma }}\right)}^{\gamma }-\left(\frac{1}{k}-1\right)\right)}^{\frac{1}{\gamma }}\right\},\right.\;\left\{1-{\left(\frac{1}{k}{\left(1-{\left(\frac{1}{C_{n}^{k}}\sum_{1\leq i_{1}<\ldots <i_{k}\leq n}{\left(1-{\left(\sum_{j=1}^{k}{\left(1-{\left(w_{i_{j}}\phi_{i_{j}}^{\gamma }-\left(w_{i_{j}}-1\right)\right)}^{\frac{1}{\gamma }}\right)}^{\gamma }-1\right)}^{\frac{1}{\gamma }}\right)}^{\gamma }+1\right)}^{\frac{1}{\gamma }}\right)}^{\gamma }-\left(\frac{1}{k}-1\right)\right)}^{\frac{1}{\gamma }}\right\}\;\left.\left\{1-{\left(\frac{1}{k}{\left(1-{\left(\frac{1}{C_{n}^{k}}\sum_{1\leq i_{1}<\ldots <i_{k}\leq n}{\left(1-{\left(\sum_{j=1}^{k}{\left(1-{\left(w_{i_{j}}\tau_{i_{j}}^{\gamma }-\left(w_{i_{j}}-1\right)\right)}^{\frac{1}{\gamma }}\right)}^{\gamma }-1\right)}^{\frac{1}{\gamma }}\right)}^{\gamma }+1\right)}^{\frac{1}{\gamma }}\right)}^{\gamma }-\left(\frac{1}{k}-1\right)\right)}^{\frac{1}{\gamma }}\right\}\right\}.$$

The detailed proof can be found in Appendix A (Proof of Theorem 7).

**Theorem** **8.** *(Monotonicity). Let* $d_{j}=\left(h_{j},g_{j},t_{j}\right)$ *and* $d_{j}^{\prime }=\left(h_{j}^{\prime },g_{j}^{\prime },t_{j}^{\prime }\right)$ *be two sets of HPFEs, if* $d_{j}\geq d_{j}^{\prime }$ *holds for all* j=1,2,…,n*, then*

(24) $$HPFSSWMSM^{\left(k\right)}\left(d_{1},d_{2},\ldots ,d_{n}\right)\geq HPFSSWMSM^{\left(k\right)}\left(d_{1}^{\prime },d_{2}^{\prime },\ldots ,d_{n}^{\prime }\right).$$

The proof of Theorem 8 is similar to that of Theorem 4, which is omitted here.

**Theorem** **9.** *(Boundedness). Let* $d_{j}=\left(h_{j},g_{j},t_{j}\right)$ (j=1,2,…,n) *be a collection of HPFEs, if* $d^{-}=\underset{j}{\min}d_{j}$ *and* $d^{+}=\underset{j}{\max}d_{j}$ *then*

(25) $$d^{-}\leq HPFSSWMSM^{\left(k\right)}\left(d_{1},d_{2},\ldots ,d_{n}\right)\leq d^{+}.$$

The proof of Theorem 9 is similar to that of Theorem 5, which is omitted here.

**Theorem** **10.** *For the given HPFEs*$d_{j}\left(j=1,2,\ldots ,n\right)$*,* k=1,2,…,n*, and their weight vector* $w={\left(w_{1},w_{2},\ldots ,w_{n}\right)}^{T}$*, the HPFSSWMSM is monotonically decreasing function with the increase in the parameter k.*

The proof is similar to the Theorem 6, and it is omitted here.

### 3.3. The Hesitant Picture Fuzzy Schweizer–Sklar Weighted Maclaurin Symmetric Mean (HPFSSWMSM) Operator

In this section, we extend the DMSM to aggregate hesitant picture fuzzy Schweizer–Sklar environment.

**Definition** **9.** *Let* $d_{j}=\left(h_{j},g_{j},t_{j}\right)$ (j=1,2,…,n) *be a collection of HPFEs, and* k=1,2,…,n*, then the operator is defined as*

(26) $$HPFSSDMSM^{\left(k\right)}\left(d_{1},d_{2},\ldots ,d_{n}\right)=\frac{1}{k}{\left(\prod_{1\leq i_{1}<\ldots <i_{k}\leq n}\sum_{j=1}^{k}d_{i_{j}}\right)}^{1/C_{n}^{k}},$$

*where* $\left(i_{1},i_{2},\ldots ,i_{k}\right)$ *traversal all the k-tuple combination of* $\left(1,2,\ldots ,n\right)$ *and* $C_{n}^{k}$ *is the binomial coefficient.*

Based on the operational laws of HPFEs, the following theorem can be obtained.

**Theorem** **11.** *Let* $d_{j}=\left(h_{j},g_{j},t_{j}\right)$ (j=1,2,…,n) *be a collection of HPFEs, and* k=1,2,…,n*, then the aggregated value by the HPFSSDMSM operator is still an HPFE and*

(27) $$HPFSSDMSM^{\left(k\right)}\left(d_{1},d_{2},\ldots ,d_{n}\right)=\cup_{\chi_{i_{j}}\in h_{i_{j}},\phi_{i_{j}}\in g_{i_{j}},\tau_{i_{j}}\in t_{i_{j}}}\;\left\{\left\{1-{\left(\frac{1}{k}{\left(1-{\left(\frac{1}{C_{n}^{k}}\sum_{1\leq i_{1}<\ldots <i_{k}\leq n}{\left(1-{\left(\sum_{j=1}^{k}{\left(1-\chi_{i_{j}}\right)}^{\gamma }-1\right)}^{\frac{1}{\gamma }}\right)}^{\gamma }+1\right)}^{\frac{1}{\gamma }}\right)}^{\gamma }-\left(\frac{1}{k}-1\right)\right)}^{\frac{1}{\gamma }}\right\},\right.\;\left\{{\left(\frac{1}{k}{\left(1-{\left(\frac{1}{C_{n}^{k}}\sum_{1\leq i_{1}<\ldots <i_{k}\leq n}{\left(1-{\left(\sum_{j=1}^{k}\phi_{i_{j}}^{\gamma }-1\right)}^{\frac{1}{\gamma }}\right)}^{\gamma }+1\right)}^{\frac{1}{\gamma }}\right)}^{\gamma }-\left(\frac{1}{k}-1\right)\right)}^{\frac{1}{\gamma }}\right\}\;\left.\left\{{\left(\frac{1}{k}{\left(1-{\left(\frac{1}{C_{n}^{k}}\sum_{1\leq i_{1}<\ldots <i_{k}\leq n}{\left(1-{\left(\sum_{j=1}^{k}\tau_{i_{j}}^{\gamma }-1\right)}^{\frac{1}{\gamma }}\right)}^{\gamma }+1\right)}^{\frac{1}{\gamma }}\right)}^{\gamma }-\left(\frac{1}{k}-1\right)\right)}^{\frac{1}{\gamma }}\right\}\right\}.$$

The proof of Theorem 11 is similar to that of Theorem 2, which is omitted here to save space.

In the following section, we discuss some desirable properties of the HPFSSDMSM operator.

**Theorem** **12.** *(Idempotency). Let* $d_{j}=d=\left(h,g,t\right)$*, for all* j=1,2,…,n*, then*

(28) $$HPFSSDMSM^{\left(k\right)}\left(d_{1},d_{2},\ldots ,d_{n}\right)=d.$$

The proof of theorem 12 is similar to that of Theorem 3, which is omitted here.

**Theorem** **13.** *(Monotonicity). Let* $d_{j}=\left(h_{j},g_{j},t_{j}\right)$ *and* $d_{j}^{\prime }=\left(h_{j}^{\prime },g_{j}^{\prime },t_{j}^{\prime }\right)$ *be two sets of HPFEs, if* $d_{j}\geq d_{j}^{\prime }$ *holds for all* j=1,2,…,n*, then*

(29) $$HPFSSDMSM^{\left(k\right)}\left(d_{1},d_{2},\ldots ,d_{n}\right)\geq HPFSSDMSM^{\left(k\right)}\left(d_{1}^{\prime },d_{2}^{\prime },\ldots ,d_{n}^{\prime }\right).$$

The proof of Theorem 13 is similar to that of Theorem 4, which is omitted here.

**Theorem** **14.** *(Boundedness). Let* $d_{j}=\left(h_{j},g_{j},t_{j}\right)$ (j=1,2,…,n) *be a collection of HPFEs, if* $d^{-}=\underset{j}{\min}d_{j}$ *and* $d^{+}=\underset{j}{\max}d_{j}$ *then*

(30) $$d^{-}\leq HPFSSDMSM^{\left(k\right)}\left(d_{1},d_{2},\ldots ,d_{n}\right)\leq d^{+}.$$

The proof of Theorem 14 is similar to that of Theorem 5, which is omitted here.

In the following, we shall discuss some special cases of the HPFSSDMSM, regarding the parameter vector *k*.

**Case** **1.** If γ→0, the HPFSSDMSM operator is reduced to hesitant picture fuzzy DMSM operator.
  (31) $$HPFSSDMSM^{\left(k\right)}\left(d_{1},d_{2},\ldots ,d_{n}\right)=\cup_{\chi_{i_{j}}\in h_{i_{j}},\phi_{i_{j}}\in g_{i_{j}},\tau_{i_{j}}\in t_{i_{j}}}\;\left\{\left\{1-{\left(1-\left({\prod_{1\leq i_{1}<\ldots <i_{k}\leq n}\left(1-\prod_{j=1}^{k}\left(1-\chi_{i_{j}}\right)\right)}^{\frac{1}{C_{n}^{k}}}\right)\right)}^{\frac{1}{k}}\right\},\right.\;\left.\left\{{\left(1-{\prod_{1\leq i_{1}<\ldots <i_{k}\leq n}\left(1-\prod_{j=1}^{k}\phi_{i_{j}}\right)}^{\frac{1}{C_{n}^{k}}}\right)}^{\frac{1}{k}}\right\},\left\{{\left(1-{\prod_{1\leq i_{1}<\ldots <i_{k}\leq n}\left(1-\prod_{j=1}^{k}\tau_{i_{j}}\right)}^{\frac{1}{C_{n}^{k}}}\right)}^{\frac{1}{k}}\right\}\right\}$$
**Case** **2.** If *k* = 1, based on the definition of HPFSSDMSM, we have
  (32) $$HPFSSDMSM^{\left(1\right)}\left(d_{1},d_{2},\ldots ,d_{n}\right)=\cup_{\chi_{i_{j}}\in h_{i_{j}},\phi_{i_{j}}\in g_{i_{j}},\tau_{i_{j}}\in t_{i_{j}}}\left\{\left\{{\left(\frac{1}{n}\sum_{i=1}^{n}\chi_{i_{j}}^{\gamma }-\frac{2}{n}+1\right)}^{\frac{1}{\gamma }}\right\},\right.\;\left.\left\{1-{\left(\frac{1}{n}\sum_{i=1}^{n}{\left(1-\phi_{i}\right)}^{\gamma }-\frac{2}{n}+1\right)}^{\frac{1}{\gamma }}\right\},\left\{1-{\left(\frac{1}{n}\sum_{i=1}^{n}{\left(1-\tau_{i}\right)}^{\gamma }-\frac{2}{n}+1\right)}^{\frac{1}{\gamma }}\right\}\right\}.$$
  In this case, the HPFSSDMSM reduces to the hesitant picture fuzzy Schweizer–Sklar geometric averaging (HPFSSGA) operator. If γ→0, the HPFSSMSM operator is reduced to hesitant picture fuzzy geometric averaging operator.
  (33) $$HPFSSDMSM^{\left(1\right)}\left(d_{1},d_{2},\ldots ,d_{n}\right)=\cup_{\chi_{i_{j}}\in h_{i_{j}},\phi_{i_{j}}\in g_{i_{j}},\tau_{i_{j}}\in t_{i_{j}}}\;\left\{\left\{{\left(\prod_{i=1}^{n}\chi_{i}\right)}^{\frac{1}{n}}\right\},\left\{1-{\left(\prod_{i=1}^{n}\left(1-\phi_{i}\right)\right)}^{\frac{1}{n}}\right\},\left\{1-{\left(\prod_{i=1}^{n}\left(1-\tau_{i}\right)\right)}^{\frac{1}{n}}\right\}\right\}.$$
**Case** **3.** If *k* = 2, based on the definition of HPFSSDMSM, we have
  (34) $$HPFSSDMSM^{\left(2\right)}\left(d_{1},d_{2},\ldots ,d_{n}\right)=\cup_{\chi_{i_{1}}\in h_{i_{1}},\chi_{i_{2}}\in h_{i_{2}},\phi_{i_{1}}\in g_{i_{1}},\phi_{i_{2}}\in g_{i_{2}},\tau_{i_{1}}\in t_{i_{1}},\tau_{i_{2}}\in t_{i_{2}}}\;\left\{\left\{1-{\left(\frac{1}{2}{\left(1-{\left(\frac{1}{n(n-1)}\sum_{\begin{array}{l} i_{1},i_{2}=1\\ i_{1}\neq i_{2} \end{array}}^{n}{\left(1-{\left({\left(1-\chi_{i_{1}}\right)}^{\gamma }+{\left(1-\chi_{i_{2}}\right)}^{\gamma }-1\right)}^{\frac{1}{\gamma }}\right)}^{\gamma }+1\right)}^{\frac{1}{\gamma }}\right)}^{\gamma }+\frac{1}{2}\right)}^{\frac{1}{\gamma }}\right\},\right.\;\left\{{\left(\frac{1}{2}{\left(1-{\left(\frac{1}{n(n-1)}\sum_{\begin{array}{l} i_{1},i_{2}=1\\ i_{1}\neq i_{2} \end{array}}^{n}{\left(1-{\left(\phi_{i_{1}}^{\gamma }+\phi_{i_{2}}^{\gamma }-1\right)}^{\frac{1}{\gamma }}\right)}^{\gamma }+1\right)}^{\frac{1}{\gamma }}\right)}^{\gamma }+\frac{1}{2}\right)}^{\frac{1}{\gamma }}\right\},\;\left.\left\{{\left(\frac{1}{2}{\left(1-{\left(\frac{1}{n(n-1)}\sum_{\begin{array}{l} i_{1},i_{2}=1\\ i_{1}\neq i_{2} \end{array}}^{n}{\left(1-{\left(\tau_{i_{1}}^{\gamma }+\tau_{i_{2}}^{\gamma }-1\right)}^{\frac{1}{\gamma }}\right)}^{\gamma }+1\right)}^{\frac{1}{\gamma }}\right)}^{\gamma }+\frac{1}{2}\right)}^{\frac{1}{\gamma }}\right\}\right\}.$$
  In this case, the HPFSSDMSM reduces to the hesitant picture fuzzy Schweizer–Sklar geometric Bonferroni mean (HPFSSGBM) operator. If γ→0, the HPFSSMSM operator is reduced to hesitant picture fuzzy geometric Bonferroni mean operator.
  (35) $$HPFSSDMSM^{\left(2\right)}\left(d_{1},d_{2},\ldots ,d_{n}\right)=\cup_{\chi_{i_{1}}\in h_{i_{1}},\chi_{i_{2}}\in h_{i_{2}},\phi_{i_{1}}\in g_{i_{1}},\phi_{i_{2}}\in g_{i_{2}},\tau_{i_{1}}\in t_{i_{1}},\tau_{i_{2}}\in t_{i_{2}}}\;\left\{\left\{1-{\left(1-{\prod_{\begin{array}{l} i_{1},i_{2}=1\\ i_{1}\neq i_{2} \end{array}}^{n}\left(1-\left(1-\chi_{i_{1}}\right)\left(1-\chi_{i_{2}}\right)\right)}^{\frac{1}{n(n-1)}}\right)}^{\frac{1}{2}}\right\},\right.\;\left.\left\{{\left(1-{\prod_{\begin{array}{l} i_{1},i_{2}=1\\ i_{1}\neq i_{2} \end{array}}^{n}\left(1-\phi_{i_{1}}\phi_{i_{2}}\right)}^{\frac{1}{n(n-1)}}\right)}^{\frac{1}{2}}\right\},\left\{{\left(1-{\prod_{\begin{array}{l} i_{1},i_{2}=1\\ i_{1}\neq i_{2} \end{array}}^{n}\left(1-\tau_{i_{1}}\tau_{i_{2}}\right)}^{\frac{1}{n(n-1)}}\right)}^{\frac{1}{2}}\right\}\right\}.$$
**Case** **4.** If *k* = 3, based on the definition of HPFSSDMSM, we have
  (36) $$HPFSSDMSM^{\left(3\right)}\left(d_{1},d_{2},\ldots ,d_{n}\right)=\cup_{\chi_{i_{1}}\in h_{i_{1}},\chi_{i_{2}}\in h_{i_{2}},\chi_{i_{3}}\in h_{i_{3}},\phi_{i_{1}}\in g_{i_{1}},\phi_{i_{2}}\in g_{i_{2}},\phi_{i_{3}}\in g_{i_{3}},,\tau_{i_{1}}\in t_{i_{1}},\tau_{i_{2}}\in t_{i_{2}},\tau_{i_{3}}\in t_{i_{3}}}\;\left\{\left\{1-{\left(\frac{1}{3}{\left(1-{\left(\sum_{i_{1},i_{2},i_{3}=1}^{n}{\left(1-{\left({\left(1-\chi_{i_{1}}\right)}^{\gamma }+{\left(1-\chi_{i_{2}}\right)}^{\gamma }+{\left(1-\chi_{i_{3}}\right)}^{\gamma }-1\right)}^{\frac{1}{\gamma }}\right)}^{\gamma }-1\right)}^{\frac{1}{\gamma }}\right)}^{\gamma }+\frac{2}{3}\right)}^{\frac{1}{\gamma }}\right\},\right.\;\left\{{\left(\frac{1}{3}{\left(1-{\left(\sum_{i_{1},i_{2},i_{3}=1}^{n}{\left(1-{\left(\phi_{i_{1}}^{\gamma }+\phi_{i_{2}}^{\gamma }+\phi_{i_{3}}^{\gamma }-1\right)}^{\frac{1}{\gamma }}\right)}^{\gamma }-1\right)}^{\frac{1}{\gamma }}\right)}^{\gamma }+\frac{2}{3}\right)}^{\frac{1}{\gamma }}\right\},\;\left.\left\{{\left(\frac{1}{3}{\left(1-{\left(\sum_{i_{1},i_{2},i_{3}=1}^{n}{\left(1-{\left(\tau_{i_{1}}^{\gamma }+\tau_{i_{2}}^{\gamma }+\tau_{i_{3}}^{\gamma }-1\right)}^{\frac{1}{\gamma }}\right)}^{\gamma }-1\right)}^{\frac{1}{\gamma }}\right)}^{\gamma }+\frac{2}{3}\right)}^{\frac{1}{\gamma }}\right\}\right\}.$$
  In this case, the HFPSSDMSM reduces to the hesitant picture fuzzy Schweizer–Sklar generalized geometric Bonferroni mean (HFPSSGGBM) operator. If γ→0, the HPFSSMSM operator is reduced to hesitant picture fuzzy generalized Bonferroni mean operator.
  (37) $$HPFSSDMSM^{\left(3\right)}\left(d_{1},d_{2},\ldots ,d_{n}\right)={\left(\sum_{i_{1},i_{2},i_{3}=1}^{n}\left(d_{i_{1}}d_{i_{2}}d_{i_{3}}\right)\right)}^{\frac{1}{3}}=\;\cup_{\chi_{i_{1}}\in h_{i_{1}},\chi_{i_{2}}\in h_{i_{2}},\chi_{i_{3}}\in h_{i_{3}},\phi_{i_{1}}\in g_{i_{1}},\phi_{i_{2}}\in g_{i_{2}},\phi_{i_{3}}\in g_{i_{3}},,\tau_{i_{1}}\in t_{i_{1}},\tau_{i_{2}}\in t_{i_{2}},\tau_{i_{3}}\in t_{i_{3}}}\;\left\{\left\{1-{\left(1-\prod_{i_{1},i_{2},i_{3}=1}^{n}\left(1-\left(1-\chi_{i_{1}}\right)\left(1-\chi_{i_{2}}\right)\left(1-\chi_{i_{3}}\right)\right)\right)}^{\frac{1}{3}}\right\},\right.\;\left.\left\{{\left(1-\prod_{i_{1},i_{2},i_{3}=1}^{n}\left(1-\phi_{i_{1}}\phi_{i_{2}}\phi_{i_{3}}\right)\right)}^{\frac{1}{3}}\right\},\left\{{\left(1-\prod_{i_{1},i_{2},i_{3}=1}^{n}\left(1-\tau_{i_{1}}\tau_{i_{2}}\tau_{i_{3}}\right)\right)}^{\frac{1}{3}}\right\}\right\}.$$
**Case** **5.** If *k* = *n*, based on the definition of HPFSSDMSM, we have
  (38) $$HPFSSDMSM^{\left(n\right)}\left(d_{1},d_{2},\ldots ,d_{n}\right)=\cup_{\chi_{i}\in h_{i},\phi_{i}\in g_{i},\tau_{i}\in t_{i}}\left\{\left\{1-{\left(\frac{1}{n}\sum_{i=1}^{n}{\left(1-\chi_{i}\right)}^{\gamma }-\frac{2}{n}+1\right)}^{\frac{1}{\gamma }}\right\},\right.\;\left.\left\{{\left(\frac{1}{n}\sum_{i=1}^{n}\phi_{i}^{\gamma }-\frac{2}{n}+1\right)}^{\frac{1}{\gamma }}\right\},\left\{{\left(\frac{1}{n}\sum_{i=1}^{n}\tau_{i}^{\gamma }-\frac{2}{n}+1\right)}^{\frac{1}{\gamma }}\right\}\right\}.$$
  In this case, the HPFSSDMSM reduces to the hesitant picture fuzzy Schweizer–Sklar averaging (HPFSSA) operator. If γ→0, the HPFSSMSM operator is reduced to hesitant picture fuzzy averaging operator.
  (39) $$HPFSSMSM^{\left(n\right)}\left(d_{1},d_{2},\ldots ,d_{n}\right)=\cup_{\chi_{i}\in h_{i},\phi_{i}\in g_{i},\tau_{i}\in t_{i}}\left\{\left\{1-{\left(\prod_{i=1}^{n}\left(1-\phi_{i}\right)\right)}^{\frac{1}{n}}\right\},\left\{{\left(\prod_{i=1}^{n}\chi_{i}\right)}^{\frac{1}{n}}\right\},\left\{{\left(\prod_{i=1}^{n}\chi_{i}\right)}^{\frac{1}{n}}\right\}\right\}.$$

**Theorem** **15.** *For the given HPFEs*$d_{j}\left(j=1,2,\ldots ,n\right)$*and* k=1,2,…,n*, the HPFSSDMSM is monotonically increasing function with the increase in the parameter k.*

The proof is similar to the Theorem 6, and it is omitted here.

### 3.4. The Hesitant Picture Fuzzy Schweizer–Sklar-Weighted Dual Maclaurin Symmetric Mean (HPFSSWDMSM) Operator

**Definition** **10.** *Let* $d_{j}=\left(h_{j},g_{j},t_{j}\right)$ (j=1,2,…,n) *be a collection of HPFEs, and* k=1,2,…,n. $w={\left(w_{1},w_{2},\ldots ,w_{n}\right)}^{T}$ *be the weight vector of* $d_{j}$*, satisfying* $w_{i}\in \left[0,1\right]$ *and* $\sum_{i=1}^{n}w_{i}=1$*, then the operator is defined as*

(40) $$HPFSSWDMSM^{\left(k\right)}\left(a_{1},a_{2},\ldots ,a_{n}\right)=\frac{1}{k}{\left(\prod_{1\leq i_{1}<\ldots <i_{k}\leq n}\sum_{j=1}^{k}\left(w_{i_{j}}a_{i_{j}}\right)\right)}^{1/C_{n}^{k}},$$

*where* $\left(i_{1},i_{2},\ldots ,i_{k}\right)$ *traversal all the k-tuple combination of* $\left(1,2,\ldots ,n\right)$*and* $C_{n}^{k}$ *is the binomial coefficient.*

According to the operations of HPFEs, the aggregated value by the HPFSSWDMSM can be obtained, which is shown as Theorem 16.

**Theorem** **16.** *Let* $d_{j}=\left(h_{j},g_{j},t_{j}\right)$ (j=1,2,…,n) *be a collection of HPFEs, and* k=1,2,…,n*, then the aggregated value by the HPFSSWDMSM operator is still an HPFE and*

(41) $$HPFSSWDMSM^{\left(k\right)}\left(a_{1},a_{2},\ldots ,a_{n}\right)=\cup_{\chi_{i_{j}}\in h_{i_{j}},\phi_{i_{j}}\in g_{i_{j}},\tau_{i_{j}}\in t_{i_{j}}}\;\left\{\left\{{\left(\frac{1}{k}{\left(1-{\left(\frac{1}{C_{n}^{k}}\sum_{1\leq i_{1}<\ldots <i_{k}\leq n}{\left(1-{\left(\sum_{j=1}^{k}{\left(1-{\left(w_{i_{j}}{\left(1-\chi_{i_{j}}\right)}^{\gamma }-\left(w_{i_{j}}-1\right)\right)}^{\frac{1}{\gamma }}\right)}^{\gamma }-1\right)}^{\frac{1}{\gamma }}\right)}^{\gamma }+1\right)}^{\frac{1}{\gamma }}\right)}^{\gamma }-\left(\frac{1}{k}-1\right)\right)}^{\frac{1}{\gamma }}\right\},\right.\;\left\{1-{\left(\frac{1}{k}{\left(1-{\left(\frac{1}{C_{n}^{k}}\sum_{1\leq i_{1}<\ldots <i_{k}\leq n}{\left(1-{\left(\sum_{j=1}^{k}{\left(1-{\left(w_{i_{j}}\phi_{i_{j}}^{\gamma }-\left(w_{i_{j}}-1\right)\right)}^{\frac{1}{\gamma }}\right)}^{\gamma }-1\right)}^{\frac{1}{\gamma }}\right)}^{\gamma }+1\right)}^{\frac{1}{\gamma }}\right)}^{\gamma }-\left(\frac{1}{k}-1\right)\right)}^{\frac{1}{\gamma }}\right\},\;\left.\left\{1-{\left(\frac{1}{k}{\left(1-{\left(\frac{1}{C_{n}^{k}}\sum_{1\leq i_{1}<\ldots <i_{k}\leq n}{\left(1-{\left(\sum_{j=1}^{k}{\left(1-{\left(w_{i_{j}}\tau_{i_{j}}^{\gamma }-\left(w_{i_{j}}-1\right)\right)}^{\frac{1}{\gamma }}\right)}^{\gamma }-1\right)}^{\frac{1}{\gamma }}\right)}^{\gamma }+1\right)}^{\frac{1}{\gamma }}\right)}^{\gamma }-\left(\frac{1}{k}-1\right)\right)}^{\frac{1}{\gamma }}\right\}\right\}.$$

The proof is similar to that of Theorem 7, which is omitted here.

**Theorem** **17.** *(Monotonicity). Let* $d_{j}=\left(h_{j},g_{j},t_{j}\right)$ *and* $d_{j}^{\prime }=\left(h_{j}^{\prime },g_{j}^{\prime },t_{j}^{\prime }\right)$ *be two sets of HPFEs, if* $d_{j}\geq d_{j}^{\prime }$ *holds for all* j=1,2,…,n*, then*

(42) $$HPFSSWDMSM^{\left(k\right)}\left(d_{1},d_{2},\ldots ,d_{n}\right)\geq HPFSSWDMSM^{\left(k\right)}\left(d_{1}^{\prime },d_{2}^{\prime },\ldots ,d_{n}^{\prime }\right).$$

The proof of Theorem 17 is similar to that of Theorem 8, which is omitted here.

**Theorem** **18.** *(Boundedness). Let* $d_{j}=\left(h_{j},g_{j},t_{j}\right)$ (j=1,2,…,n) *be a collection of HPFEs, if* $d^{-}=\underset{j}{\min}d_{j}$ *and* $d^{+}=\underset{j}{\max}d_{j}$ *then*

(43) $$d^{-}\leq HPFSSWDMSM^{\left(k\right)}\left(d_{1},d_{2},\ldots ,d_{n}\right)\leq d^{+}.$$

The proof of Theorem 18 is similar to that of Theorem 9, which is omitted here.

## 4. A Novel Approach to MAGDM Based on the Proposed Operators

In this section, we shall apply the proposed aggregation operators to solving MAGDM problems in hesitant picture fuzzy environment. Let $A=\left\{A_{1},A_{2},\ldots ,A_{m}\right\}$ be a set of alternatives, and $G=\left\{G_{1},G_{2},\ldots ,G_{n}\right\}$ be a set of attributes. The weight vector of attributes is $w={\left(w_{1},w_{2},\ldots ,w_{n}\right)}^{T}$. A group of decision makers is responsible for the evaluation of each alternative under each attribute. The evaluation results of attribute $G_{j}\left(j=1,2,\ldots ,n\right)$ of alternative $A_{i}\left(i=1,2,\ldots ,m\right)$ are denoted by an HPFE $d_{ij}=\left\{\left\{h_{ij}\right\},\left\{g_{ij}\right\}\right\}$. The decision-making method consists of the following steps.

**Step** **1.** Normalize the original decision matrix. Generally, attributes can be divided into two types: benefit type and cost type. Therefore, the original decision matrix should be normalized by

(44) $$d_{ij}=\left\{\begin{array}{l} \left\{\left\{h_{ij}\right\},\left\{g_{ij}\right\}\right\}\;G_{j}\in I_{1}\\ \left\{\left\{g_{ij}\right\},\left\{h_{ij}\right\}\right\}\;G_{j}\in I_{2} \end{array}\right.$$

where $I_{1}$ and $I_{2}$ represent the benefit type and cost type of attributes, respectively.

**Step** **2.** Utilize HPFWMSM operator

(45) $$d_{i}=HPFWMSM^{\left(k\right)}\left(d_{i1},d_{i2},\ldots ,d_{in}\right)$$

or

(46) $$d_{i}=HPFWDMSM^{\left(k\right)}\left(d_{i1},d_{i2},\ldots ,d_{in}\right),$$

to aggregate the decision makers’ preference information and calculate the overall values of alternatives.

**Step** **3.** Rank $d_{i}\left(i=1,2,\ldots ,m\right)$ according to Definition 3 and select the best alternative accordingly.

## 5. The Application of the Proposed Method to Enterprise Informatization Level Evaluation

**Example** **4.** *In the information age, the operation and development of enterprise are inseparable from information construction. Enterprise information construction aims to improve the management level of the entire company through the computer science and information technology. Enterprises can improve the efficiency of their operations and management by setting up the information director, equipping with automatic and intelligent high-tech hardware and software equipment and facilities, establishing a work platform including network databased on all kinds of information management systems, to meet the requirement of modern enterprise management. In general, it is necessary to make an informatization level assessment before the formal implementation of enterprise informatization construction. In reality, DMs should provide their evaluations for the enterprise with respect to a set of attributes; thus, the evaluation process can be regarded as a typical MAGDM problem. Suppose that a group is planning to help its four subsidiaries to improve their informatization construction level. In line with the principle of maximum resource utilization, the group decided to invite three experts to make information level evaluation for the four subsidiaries* $A_{i}\left(i=1,2,3,4\right)$*with respect to four attributes* $G_{i}\left(i=1,2,3,4\right)$*. Considering the availability of information and the feasibility of the survey, four attributes are ultimately selected: G1 (IT coverage rate), G2 (IT resource coverage rate), G3 (IT contribution rate), and G4 (proportion of investment for informatization). The weight vector of three experts is* $\varpi ={\left(0.2,0.3,0.5\right)}^{T}$*, and the weight vector of four attributes is* $w={\left(0.25,0.35,0.15,0.25\right)}^{T}$*. Taking into account the complexity of real-world scenarios and the accessibility of information, the final decision maker requires DMs to employ hesitant picture fuzzy elements to express their evaluation information. Therefore, the decision matrices given by DMs are shown in* Table 1, Table 2 and Table 3.

### 5.1. Enterprise Informatization Level Evaluation Process with HPFSSWMSM Operator

**Step** **1.** According to the case description, all attributes are classified as positive and therefore no need to be normalized.
**Step** **2.** We know that the parameter variable (PV) *k* plays an important role in considering the interrelationship among the input arguments. As for the DMs in this example are independent among each other, it is appropriate to take *k* = 1 as the value of PV. Then, the HPFSSWMSM operator reduces to the weighted form of Equation (47), that is, hesitant picture fuzzy Schweizer–Sklar-weighted averaging (HPFSSWA) operator:
  (47) $$HPFSSWMSM^{\left(1\right)}\left(d_{1},d_{2},\ldots ,d_{n}\right)=\frac{1}{n}\left(\sum_{i=1}^{n}w_{i}d_{i}\right)=\cup_{\chi_{i}\in h_{i},\phi_{i}\in g_{i},\tau_{i}\in t_{i}}\;\left\{\left\{1-{\left(\frac{1}{n}\left(\sum_{i=1}^{n}\left(w_{i}{\left(1-\chi_{i}\right)}^{\gamma }-\left(w_{i}-1\right)\right)-1\right)-\left(\frac{1}{n}-1\right)\right)}^{1/\gamma }\right\},\right.\;\left\{{\left(\frac{1}{n}\left(\sum_{i=1}^{n}\left(w_{i}\phi_{i}^{\gamma }-\left(w_{i}-1\right)\right)-1\right)-\left(\frac{1}{n}-1\right)\right)}^{1/\gamma }\right\},\;\left.\left\{{\left(\frac{1}{n}\left(\sum_{i=1}^{n}\left(w_{i}\tau_{i}^{\gamma }-\left(w_{i}-1\right)\right)-1\right)-\left(\frac{1}{n}-1\right)\right)}^{1/\gamma }\right\}\right\}.$$
  Compute the overall comprehensive assessments $d_{ij}^{\prime }=\left\{\left\{h_{ij}^{\prime }\right\},\left\{g_{ij}^{\prime }\right\},\left\{t_{ij}^{\prime }\right\}\right\}$ of alternatives $A_{i}\left(i=1,2,\ldots ,m\right)$ with respect to $G_{j}\left(j=1,2,\ldots ,n\right)$. According to Equation (47) (suppose that γ=−1), we can obtain the comprehensive evaluations, which is shown in Appendix A (Comprehensive matrix of subsection 5.1).
**Step** **3.** Compute the global evaluation values with the proposed HPFSSWMSM operator (suppose that k=2,γ=−1) and the scores of alternatives with Definition 3, we can derive
  $$s\left(d_{1}\right)=-0.8480,s\left(d_{2}\right)=-0.7705,s\left(d_{3}\right)=-0.8018,s\left(d_{4}\right)=-0.8190$$
  According to the scores of alternatives, the ranking order is $A_{2}≻A_{3}≻A_{4}≻A_{1}$. Therefore, $A_{2}$ is the best alternative.

### 5.2. Enterprise Informatization Level Evaluation Process with HPFSSWDMSM Operator

**Step** **1.** The step 1 of decision-making process with HPFSSWDMSM operator is same as the above.
**Step** **2.** For the same reason as the above, we assign *k* = 1 to the HPFSSWDMSM operator, which can make it reduce to the weighted form of Equation (48), that is, hesitant picture fuzzy Schweizer–Sklar-weighed geometric (HPFSSWG) operator:
  (48) $$HPFSSWDMSM^{\left(1\right)}\left(d_{1},d_{2},\ldots ,d_{n}\right)={\left(\prod_{i=1}^{n}{\left(d_{i}\right)}^{w_{i}}\right)}^{\frac{1}{n}}=\cup_{\chi_{i}\in h_{i},\phi_{i}\in g_{i},\tau_{i}\in t_{i}}\;\left\{\left\{{\left(\frac{1}{n}\left(\sum_{i=1}^{n}\left(w_{i}\chi^{\gamma }-\left(w_{i}-1\right)\right)-1\right)-\left(\frac{1}{n}-1\right)\right)}^{1/\gamma }\right\},\right.\left\{1-{\left(\frac{1}{n}\left(\sum_{i=1}^{n}\left(w_{i}{\left(1-\phi \right)}^{\gamma }-\left(w_{i}-1\right)\right)-1\right)-\left(\frac{1}{n}-1\right)\right)}^{1/\gamma }\right\},\;\left.\left\{1-{\left(\frac{1}{n}\left(\sum_{i=1}^{n}\left(w_{i}{\left(1-\tau \right)}^{\gamma }-\left(w_{i}-1\right)\right)-1\right)-\left(\frac{1}{n}-1\right)\right)}^{1/\gamma }\right\}\right\}.$$
  Compute the overall comprehensive assessments $d_{ij}^{\prime }=\left\{\left\{h_{ij}^{\prime }\right\},\left\{g_{ij}^{\prime }\right\},\left\{t_{ij}^{\prime }\right\}\right\}$ of alternatives $A_{i}\left(i=1,2,\ldots ,m\right)$ with respect to $G_{j}\left(j=1,2,\ldots ,n\right)$. According to Equation (48) (suppose that γ=−1), then we can obtain the comprehensive evaluations, which is shown in Appendix A (Comprehensive matrix of subsection 5.2).
**Step** **3.** Compute the global evaluation values with the proposed HPFSSWDMSM operator (suppose that k=2,γ=−1) and the scores of alternatives with Definition 3, we can derive
  $$s\left(d_{1}\right)=-0.8802,s\left(d_{2}\right)=-0.8059,s\left(d_{3}\right)=-0.8267,s\left(d_{4}\right)=-0.8320$$
  According to the scores of alternatives, the ranking order is $A_{2}≻A_{3}≻A_{4}≻A_{1}$. Therefore, $A_{2}$ is the best alternative.

### 5.3. Parameter Analysis

The proposed method contains two parameters, *k* and *γ*, which definitely affect the final result. To illustrate the functions of these two parameters, we assign different values to *k* and *γ* to solve the Example 1, the results and detail explanations are shown in the following section.

#### 5.3.1. The Influence of *k*

Bulleted lists look like this: As mentioned above, the PV *k* of the proposed method is of great significance in the information aggregation process. With the PV, the proposed method can not only realize different functions by assigning different values to k but can also degenerate into other simpler operators in some special cases. To give a more explanatory and persuasive illustration, we provide several calculation results with some different values of the PV *k*, which is shown in Table 4.

**Figure 1:**
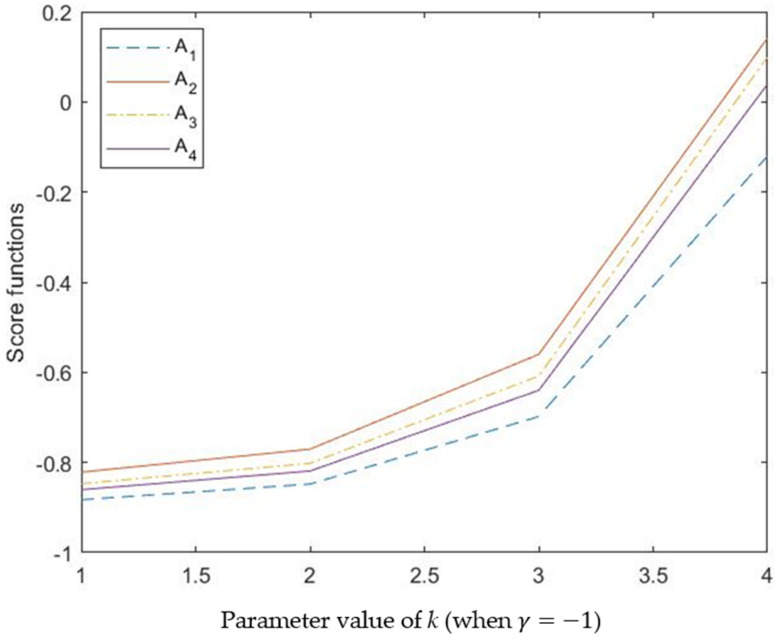
Score functions calculated by different values of PV *k* when γ=−1.

As we can see from Table 4, the score functions of alternatives $A_{i}\left(i=1,2,3,4\right)$ changes with the value of PV *k*, and the scores become much higher when the value of *k* becomes much larger, which indicates that the PV *k* indeed has an influence on the decision-making result. Figure 1 clearly shows this characteristic in a visual form. The PV *k* can improve the flexibility of the method because that value of *k* represents the number of attributes that have interrelationships, and *k* = 4 means that there are no independent attributes and all of them are in a codependent relationship, which promotes alternative *A*_1_ but inhibits *A*_4_. The slight changes of ranking results proves that the PV *k* has an effective influence on the decision making. In practical applications, it should be noted that the value of PV *k* should be considered according to the actual situation.

#### 5.3.2. The Influence of *γ*

**Figure 2:**
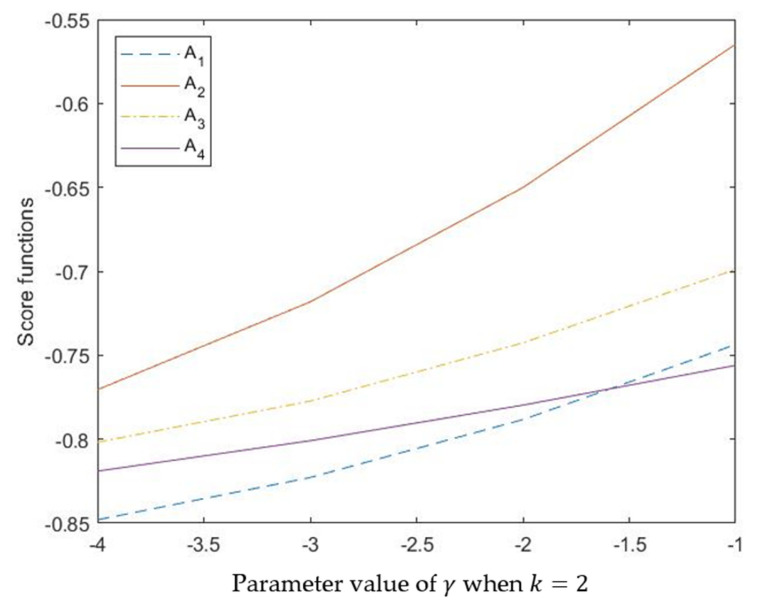
Score functions calculated by different values of PV *k* when γ=−1.

The parameter *γ* also has some influences on the aggregation result. We know that parameter *γ* is a negative real number and the Schweizer–Sklar *t*-norm and *t*-conorm reduces to the algebraic *t*-norm and algebraic *t*-conorm when γ→0. To further explore its role in information aggregation, this subsection provides additional experiments for the changes of the calculation results when *γ* takes different values, the experimental results are shown in Table 5, and the visual graph is shown in Figure 2.

As we can see from Table 4, the score functions of alternatives $A_{i}\left(i=1,2,3,4\right)$ change with the value of PV *k*, and the scores become much higher when the value of *k* becomes much larger, which indicates that the PV *k* indeed has an influence on the decision-making result. Figure 1 clearly shows this characteristic in a visual form. The PV *k* can improve the flexibility of the method because that value of *k* represents the number of attributes that have interrelationships, and *k* = 4 means that there are no independent attributes and all of them are in a codependent relationship, which promotes alternative *A*_1_ but inhibits *A*_4_. The slight changes of ranking results prove that the PV *k* has an effective influence on decision making. In practical applications, it should be noted that the value of PV *k* should be considered according to the actual situation.

### 5.4. Validity Analysis

To show the validity and effectiveness of our method, we utilize it and Wang and Liu’s [33] method based on intuitionistic fuzzy Schweizer–Sklar-weighed MSM (IFSSWMSM) operator and Biswas and Deb’s [37] method based on Pythagorean fuzzy Schweizer–Sklar power-weighed aggregation (PFSSPWA) operator to solve the numerical example in Reference [33] (details can be found in section of Reference [33]), and the calculation results are shown in Table 6.

The original data of the numerical example in Reference [33] are expressed in intuitionistic fuzzy number with a MD and an NMD. It is entirely appropriate to utilize the PFSSPWA operator [37] to process this batch of data. However, the HPFE is expressed with a PMD, an NLMD, and an NEMD. To enable the proposed operator effectively applied to this case, we set all the NLMDs to 0, so that the information contained in the original data is not affected and our proposed method can be well matched. As can be seen from Table 6, the results obtained by the four methods are the same, e.g., $A_{2}≻A_{3}≻A_{4}≻A_{5}≻A_{1}$, which demonstrates that our method proposed in this paper is valid and effective in dealing with these kinds of multiple-attribute decision-making problems. It is noted that the score functions obtained by Wang and Liu’s [33] method $\left(k=1,\gamma =-2\right)$ is the same as that obtained by our method with HPFSSWMSM operator $\left(k=1,\gamma =-2\right)$. In fact, when all the NLMDs take the value 0 and there is only one element in PMD and NEMD, the proposed HPFSSWMSM operator degenerates into IFSSWMSM operator [33], as we discussed in Remark 1. Therefore, it is not surprising that the two methods can reach exactly the same conclusions, which from the other side proves our method is more flexible and more applicable.

### 5.5. Comparative Analysis

To further illustrate the merits and superiorities of the proposed method, we conduct some comparative analysis. The methods utilized to participate in the comparison include: Wang and Liu’s [33] method based on IFSSWMSM operator, Wei’s [38] method based on picture fuzzy weighted average (PFWA) operator, and Wang et al.’s [39] method based on picture fuzzy weighted Muirhead mean (PFWMM) operators.

The fuzzy information that different fuzzy sets can handle is different, and the most basic is IFS. To make all the fuzzy sets participating in the comparison be effectively involved in making decisions, we decided to employ the example of Wang and Liu [33] for comparative analysis, because their method is based on IFSSWMSM operator which is built on top of IFS. In the following, we give a briefly description of the example.

**Example** **5.** **(Adopted from Reference** [33]**).** *Consider an investment problem, which aims to choose the best company from five possible companies* $A_{i}\left(i=1,2,3,4,5\right)$*; three DMs* $M_{r}\left(r=1,2,3\right)$ *with the weight of* $\varpi ={\left(0.35,0.4,0.25\right)}^{T}$ *are invited and required to provide their evaluations by the intuitionistic fuzzy numbers with respect to four* attributes $G_{j}\left(j=1,2,3,4\right)$*, including the risk factor* $G_{1}$*, the growth factor* $G_{2}$*, the social-political impact* $G_{3}$*, and the environmental impact* $G_{4}$*. The weight of the attributes is* $w={\left(0.2,0.1,0.3,0.4\right)}^{T}$*. We show the decision matrix of the second DM* $D^{2}={\left(d_{ij}^{2}\right)}_{5\times 4}$ *in* Table 7*, and the rest of which can be found in Reference* [33].

The same as the previous analysis, to enable the proposed operator effectively applied to this case, we make some small changes to the original data by setting all the NLMDs to 0 without changing the information contained in them (the adjusted data of Table 7 in shown in Table 8). The methods in [38,39] are based on PFS, which also consists of a PMD and an NEMD. Therefore, there is no need to worry about the case suitability. After calculation, the results obtained from these methods are shown in Table 9.

From Example 2, we can easily find that the attributes are interdependent and have interrelationships with each other. Table 9 shows the score values and ranking orders of all operators participating in the comparative analysis. We can see that ranking orders of Wei’s [38] method based on PFWA operator and the proposed method based on HPFSSWMSM operator when k=1,γ→0 are the same. This is because the weighted Maclaurin symmetric mean operator can reduce to weighted average operator when *k* = 1, the SSTT operational rules can reduce to ATT when γ→0, and the hesitant picture fuzzy set can be regarded as a special kind of Pythagorean fuzzy set when the elements of NLMD are 0. In other words, Wei’s [38] method can be deemed a degenerate case of the proposed method. By comparison, the ranking result of our method with HPFSSWMSM operator when k=1,γ=−2 is different from Wei’s [38] method because the parameter *γ* plays a role in the final result.

In addition, ranking orders of Wang et al.’s [39] method based on PFWMM $\left(P=\left[1,1,1,1\right]\right)$ is the same as our proposed method when the PV *k* takes values of 2, 3 and 4. We have known that our methods have the ability to capture the possible interrelationships from multiple attributes, and similarly, the fusion operator weighted Muirhead mean of Wang et al.’s [39] method also possesses this skill with the PV *P*. Under the premise of considering the interaction, the two methods have reached the same conclusion. As for Wang and Liu’s [33] method based on the IFSSWMSM operator, it has the same information fusion operator just as our proposed method, the difference is that in our method DMs utilize HPFEs to express their assessments while Wang and Liu’s [33] method utilizes the intuitionistic fuzzy numbers. However, in this case, the original data are adjusted so that both fuzzy sets can be applied to solve it, and the priority of the HPFS cannot be incarnated. To further highlight the advantages of this point, we further adjust the data in Table 8 into the following form, shown in Table 10.

Formally, we can divide the data in Table 10 into two types: one can be degenerated into intuitionistic fuzzy numbers, such as $d_{22}^{2}=\left\{\left\{0.6\right\},\left\{0\right\},\left\{0.2\right\}\right\}$; the other one cannot do that, such as $d_{52}^{2}=\left\{\left\{0.7\right\},\left\{0.05\right\},\left\{0.2\right\}\right\}$. When combined with the conversion from Table 7 and Table 8, we can find that the intuitionistic fuzzy numbers can be easily expanded to HPFEs, but the reverse is difficult to achieve. Therefore, our proposed method is much more common than Wang and Liu’s [33] model and can be applied to many scenarios.

In the following section, we summarize the results of the comparative analysis as follows:

1. Compared with Wei’s [38] method, our proposed method is more generic and flexible. Wei’s method is based on the PFWA operator, which consists of the Pythagorean fuzzy sets and basically the most traditional weighted average operator. Both the information expression method and the information aggregation method of Wei’s [38] basic operator is only suitable for simple and independent general fuzzy decision-making problems. The information aggregation method is based on the premise that the input variables are independent of each other. This harsh assumption severely limits the scope of application of this method. In real-life decision-making problems, it is common that the evaluation criteria are interdependent and are related to each other. Therefore, our method with the MSM operator as the information fusion operator has much stronger practical application value. Because of that, by assigning different values to the PV *k*, our method can handle several kinds of situations with complex relationships.
2. Compared with Wang et al.’s [39] method, our proposed method can be employed in a wider range. Wang et al.’s [39] method is based on the PFS just as Wei’s [38] method, and it allows the square sum of the PMD and the NEMD to be no larger than one, which is a strict condition for DMs to spend time and energy to confirm their evaluations. The proposed method gives DMs the greatest tolerance, allowing them to provide several possible values of their uncertain information with nothing to worry about. On the other hand, Wang et al.’s [39] method is based on the traditional operational rules of ATT, while our method is based on the new operational rules of SSTT, which is more flexible and can take the risk preference of DMs into consideration by the parameter *γ*.
3. Compared with Wang and Liu’s [33] method, our proposed method provides DMs more freedom when they feel hesitant or uncertain among several evaluation values and thus more adaptive at coping with the MAGDM problems with a high degree of complexity and more friendly to the qualitative problems that rely on the subjective decision making of DMs. These advantages are due to the fact that the information expression module of our method is based on the hesitant picture fuzzy set, whose elements are constructed with a PMD, an NLMD, and an NEMD, with which the HPFEs are able to tolerate the ambiguity of DMs’ assessments to the greatest extent.

In the following section, we summarize the comparison of the proposed method with Wang and Liu’s [33], Wei’s [38], and Wang et al.’s [39] methods in Table 11.

## 6. Conclusions

The main purpose of this paper was to present a novel MAGDM problem and apply it to a real enterprise informatization level evaluation problem. To achieve this goal, we mainly focused on three parts: information expression method, information aggregation method, and the operational rules in the process of information aggregation. Firstly, we proposed the concept of HPFS, which is an effective and powerful information expression method in coping with uncertainty. The advantage of the proposed HPFS is that it can provide DMs more freedom with several possible elements in PMD, NLMD, and NEMD. Furthermore, the Schweizer–Sklar *t*-norm and *t*-conorm operational rules for HPFEs were proposed to provide a more flexible computing environment. In order to capture the interrelationship among attributes, we employed the MSM operator to aggregate evaluation information with HPFEs and introduced a series of operators combining HPFS and MSM under which SSTT operational rules were introduced, that is, the HPFSSMSM operator, HPFSSWMSM operator, HPFSSDMSM operator, and HPFSSWDMSM operator. With the proposed operator, the interrelationship among different attributes could be taken into consideration. Then, a novel approach to MAGDM problems based on the proposed operators was given. Finally, we applied the proposed method to an enterprise informatization level evaluation problem, and the HPFSSWMSM and HPFSSWDMSM operators were utilized to solve the problem, respectively. To illustrate the advantages of the proposed method, we utilized it to compare with some existing methods, and the results showed that our method is more flexible, more effective, and more liberal for decision makers. In future works, we will explore the application possibility of more algorithms in hesitant picture fuzzy sets and more aggregation operators, such as the Muirhead mean operator and power average operator also being worth exploring. In addition, our proposed method can be applied to more areas, such as the supplier selection problem, investment issues, etc. The comparison between the proposed method and some traditional methods can be conducted to show the validity.

## Figures and Tables

**Table 1:** The evaluation values given by DM *D*_1_.

	*G*_1_	*G*_2_	*G*_3_	G_4_
*A_1_*	$\left(\left\{0.1,0.2\right\},\left\{0.5\right\},\left\{0.3,0.5\right\}\right)$	$\left(\left\{0.2\right\},\left\{0.5\right\},\left\{0.2,0.3\right\}\right)$	$\left(\left\{0.3,0.4\right\},\left\{0.1,0.2\right\},\left\{0.4\right\}\right)$	$\left(\left\{0.09,0.12\right\},\left\{0.13\right\},\left\{0.66,0.71\right\}\right)$
*A_2_*	$\left(\left\{0.14,0.23\right\},\left\{0.03\right\},\left\{0.65,0.7\right\}\right)$	$\left(\left\{0.03,0.34\right\},\left\{0.2\right\},\left\{0.44\right\}\right)$	$\left(\left\{0.12,0.34\right\},\left\{0.02\right\},\left\{0.44,0.5\right\}\right)$	$\left(\left\{0.06\right\},\left\{0.04\right\},\left\{0.78,0.85\right\}\right)$
*A_3_*	$\left(\left\{0.25\right\},\left\{0.08\right\},\left\{0.64\right\}\right)$	$\left(\left\{0.18,0.22\right\},\left\{0.03\right\},\left\{0.6,0.67\right\}\right)$	$\left(\left\{0.13,0.21\right\},\left\{0.03,0.06\right\},\left\{0.72\right\}\right)$	$\left(\left\{0.04,0.06\right\},\left\{0.08,0.12\right\},\left\{0.8\right\}\right)$
*A_4_*	$\left(\left\{0.09,0.13\right\},\left\{0.02\right\},\left\{0.76\right\}\right)$	$\left(\left\{0.02,0.05\right\},\left\{0.02\right\},\left\{0.68\right\}\right)$	$\left(\left\{0.13\right\},\left\{0.15\right\},\left\{0.65\right\}\right)$	$\left(\left\{0.14,0.23\right\},\left\{0.05\right\},\left\{0.55,0.68\right\}\right)$

**Table 2:** The evaluation values given by DM *D*_2_.

Tab	*G*_1_	*G*_2_	*G*_3_	*G*_4_
*A*_1_	$\left(\left\{0.06\right\},\left\{0.06\right\},\left\{0.76,0.86\right\}\right)$	$\left(\left\{0.07\right\},\left\{0.2,0.23\right\},\left\{0.55\right\}\right)$	$\left(\left\{0.13,0.24\right\},\left\{0.01,0.02\right\},\left\{0.73\right\}\right)$	$\left(\left\{0.05,0.08\right\},\left\{0.1\right\},\left\{0.7,0.81\right\}\right)$
*A*_2_	$\left(\left\{0.23\right\},\left\{0.12\right\},\left\{0.62\right\}\right)$	$\left(\left\{0.45,0.5\right\},\left\{0.05\right\},\left\{0.2\right\}\right)$	$\left(\left\{0.3,0.34\right\},\left\{0.2\right\},\left\{0.24\right\}\right)$	$\left(\left\{0.2\right\},\left\{0.03\right\},\left\{0.6,0.73\right\}\right)$
*A*_3_	$\left(\left\{0.02\right\},\left\{0.01\right\},\left\{0.82,0.91\right\}\right)$	$\left(\left\{0.52\right\},\left\{0.02\right\},\left\{0.43\right\}\right)$	$\left(\left\{0.22,0.24\right\},\left\{0.06\right\},\left\{0.64\right\}\right)$	$\left(\left\{0.34\right\},\left\{0.12\right\},\left\{0.5\right\}\right)$
*A*_4_	$\left(\left\{0.04,0.07\right\},\left\{0.05\right\},\left\{0.76,0.81\right\}\right)$	$\left(\left\{0.55\right\},\left\{0.03\right\},\left\{0.4\right\}\right)$	$\left(\left\{0.05\right\},\left\{0.03\right\},\left\{0.81\right\}\right)$	$\left(\left\{0.02,0.05\right\},\left\{0.02\right\},\left\{0.68\right\}\right)$

**Table 3:** The evaluation values given by DM *D*_3_.

	*G*_1_	*G*_2_	*G*_3_	*G*_4_
*A*_1_	$\left(\left\{0.1\right\},\left\{0.01\right\},\left\{0.66,0.72\right\}\right)$	$\left(\left\{0.04\right\},\left\{0.2\right\},\left\{0.6,0.73\right\}\right)$	$\left(\left\{0.04\right\},\left\{0.17,0.2\right\},\left\{0.64\right\}\right)$	$\left(\left\{0.08\right\},\left\{0.04\right\},\left\{0.72,0.8\right\}\right)$
*A*_2_	$\left(\left\{0.3\right\},\left\{0.1\right\},\left\{0.6\right\}\right)$	$\left(\left\{0.23,0.25\right\},\left\{0.02\right\},\left\{0.52,0.6\right\}\right)$	$\left(\left\{0.2,0.25\right\},\left\{0.1\right\},\left\{0.62\right\}\right)$	$\left(\left\{0.13\right\},\left\{0.04\right\},\left\{0.78\right\}\right)$
*A*_3_	$\left(\left\{0.2\right\},\left\{0.01,0.04\right\},\left\{0.72\right\}\right)$	$\left(\left\{0.38,0.42\right\},\left\{0.09\right\},\left\{0.43\right\}\right)$	$\left(\left\{0.15,0.2\right\},\left\{0.18\right\},\left\{0.67\right\}\right)$	$\left(\left\{0.18,0.22\right\},\left\{0.07\right\},\left\{0.62\right\}\right)$
*A*_4_	$\left(\left\{0.07\right\},\left\{0.23\right\},\left\{0.64\right\}\right)$	$\left(\left\{0.23\right\},\left\{0.12\right\},\left\{0.62\right\}\right)$	$\left(\left\{0.2\right\},\left\{0.03\right\},\left\{0.6,0.73\right\}\right)$	$\left(\left\{0.09,0.13\right\},\left\{0.02\right\},\left\{0.76\right\}\right)$

**Table 4:** The calculation results by different values of *k* with HPFSSWMSM operator (γ=−1).

k	$\mathrm{Score}\;\mathrm{Function}\;s\left(d_{i}\right)\left(i=1,2,3,4\right)$	Ranking Orders
*k* = 1	$s\left(d_{1}\right)=-0.8828$, $s\left(d_{2}\right)=-0.8215$, $s\left(d_{3}\right)=-0.8471$, $s\left(d_{4}\right)=-0.8605$	$A_{2}≻A_{3}≻A_{4}≻A_{1}$
*k* = 2	$s\left(d_{1}\right)=-0.8480$, $s\left(d_{2}\right)=-0.7705$, $s\left(d_{3}\right)=-0.8018$, $s\left(d_{4}\right)=-0.8190$	$A_{2}≻A_{3}≻A_{4}≻A_{1}$
*k* = 3	$s\left(d_{1}\right)=-0.6981$, $s\left(d_{2}\right)=-0.5600$, $s\left(d_{3}\right)=-0.6083$, $s\left(d_{4}\right)=-0.6399$	$A_{2}≻A_{3}≻A_{4}≻A_{1}$
*k* = 4	$s\left(d_{1}\right)=-0.1199$, $s\left(d_{2}\right)=0.1416$, $s\left(d_{3}\right)=0.0996$, $s\left(d_{4}\right)=0.0394$	$A_{2}≻A_{3}≻A_{4}≻A_{1}$

**Table 5:** The calculation results by different values of *𝛾* with HPFSSWMSM operator (k=2).

𝛾	$\mathrm{Score}\;\mathrm{Function}\;s\left(d_{i}\right)\;\left(i=1,2,3,4\right)$	Ranking Orders
𝛾 = −1	$s\left(d_{1}\right)=-0.8480$, $s\left(d_{2}\right)=-0.7705$, $s\left(d_{3}\right)=-0.8018$, $s\left(d_{4}\right)=-0.8190$	$A_{2}≻A_{3}≻A_{4}≻A_{1}$
𝛾 = −2	$s\left(d_{1}\right)=-0.8228$, $s\left(d_{2}\right)=-0.7182$, $s\left(d_{3}\right)=-0.7773$, $s\left(d_{4}\right)=-0.8009$	$A_{2}≻A_{3}≻A_{4}≻A_{1}$
𝛾 = −3	$s\left(d_{1}\right)=-0.7883$, $s\left(d_{2}\right)=-0.6501$, $s\left(d_{3}\right)=-0.7426$, $s\left(d_{4}\right)=-0.7797$	$A_{2}≻A_{3}≻A_{4}≻A_{1}$
𝛾 = −4	$s\left(d_{1}\right)=-0.7436$, $s\left(d_{2}\right)=-0.5647$, $s\left(d_{3}\right)=-0.6988$, $s\left(d_{4}\right)=-0.7560$	$A_{2}≻A_{3}≻A_{1}≻A_{4}$

**Table 6:** The calculation results by different methods.

Methods	$\mathrm{Score}\;\mathrm{Function}\;s\left(d_{i}\right)\left(i=1,2,3,4\right)$	Ranking Orders
Wang and Liu’s [33] method based on IFSSWMSM operator $\left(k=1,\gamma =-2\right)$	$s\left(d_{1}\right)=-0.6704$, $s\left(d_{2}\right)=-0.1488$, $s\left(d_{3}\right)=-0.2831$, $s\left(d_{4}\right)=-0.4569$, $s\left(d_{5}\right)=-0.6489$	$A_{2}≻A_{3}≻A_{4}≻A_{5}≻A_{1}$
Biswas and Deb’s [37] method based on PFSSPWA operator $\left(\eta =-2\right)$	$s\left(d_{1}\right)=0.4876,$ $s\left(d_{2}\right)=0.8263,$ $s\left(d_{3}\right)=0.6308,$ $s\left(d_{4}\right)=0.5659,$ $s\left(d_{5}\right)=0.5324$	$A_{2}≻A_{3}≻A_{4}≻A_{5}≻A_{1}$
$\mathrm{Our}\;\mathrm{method}\;\mathrm{with}\;\mathrm{HPFSSWMSM}\;\mathrm{operator}\;\left(k=1,\gamma =-2\right)$	$s\left(d_{1}\right)=-0.6704,$ $s\left(d_{2}\right)=-0.1488,$ $s\left(d_{3}\right)=-0.2831,$ $s\left(d_{4}\right)=-0.4569,$ $s\left(d_{5}\right)=-0.6489$	$A_{2}≻A_{3}≻A_{4}≻A_{5}≻A_{1}$
$\mathrm{Our}\;\mathrm{method}\;\mathrm{with}\;\mathrm{HPFSSWDMSM}\;\mathrm{operator}\left(k=1,\gamma =-2\right)$	$s\left(d_{1}\right)=-0.5131,$ $s\left(d_{2}\right)=0.0925,$ $s\left(d_{3}\right)=-0.0890,$ $s\left(d_{4}\right)=-0.2755,$ $s\left(d_{5}\right)=-0.4675$	$A_{2}≻A_{3}≻A_{4}≻A_{5}≻A_{1}$

**Table 7:** The evaluation values of Example 2 given by DM *D*_2_.

	*G*_1_	*G*_2_	*G*_3_	*G*_4_
*A*_1_	$\left(0.4,0.5\right)$	$\left(0.6,0.2\right)$	$\left(0.5,0.4\right)$	$\left(0.5,0.3\right)$
*A*_2_	$\left(0.5,0.4\right)$	$\left(0.6,0.2\right)$	$\left(0.6,0.3\right)$	$\left(0.7,0.3\right)$
*A*_3_	$\left(0.4,0.5\right)$	$\left(0.3,0.5\right)$	$\left(0.4,0.4\right)$	$\left(0.2,0.6\right)$
*A*_4_	$\left(0.5,0.4\right)$	$\left(0.7,0.2\right)$	$\left(0.4,0.4\right)$	$\left(0.6,0.2\right)$
*A*_5_	$\left(0.6,0.3\right)$	$\left(0.7,0.2\right)$	$\left(0.4,0.2\right)$	$\left(0.7,0.2\right)$

**Table 8:** The adjusted evaluation values of Example 2 given by DM *D*_2_.

	*G*_1_	*G*_2_	*G*_3_	*G*_4_
*A*_1_	$\left\{\left\{0.4\right\},\left\{0\right\},\left\{0.5\right\}\right\}$	$\left\{\left\{0.6\right\},\left\{0\right\},\left\{0.2\right\}\right\}$	$\left\{\left\{0.5\right\},\left\{0\right\},\left\{0.4\right\}\right\}$	$\left\{\left\{0.5\right\},\left\{0\right\},\left\{0.3\right\}\right\}$
*A*_2_	$\left\{\left\{0.5\right\},\left\{0\right\},\left\{0.4\right\}\right\}$	$\left\{\left\{0.6\right\},\left\{0\right\},\left\{0.2\right\}\right\}$	$\left\{\left\{0.6\right\},\left\{0\right\},\left\{0.3\right\}\right\}$	$\left\{\left\{0.7\right\},\left\{0\right\},\left\{0.3\right\}\right\}$
*A*_3_	$\left\{\left\{0.4\right\},\left\{0\right\},\left\{0.5\right\}\right\}$	$\left\{\left\{0.3\right\},\left\{0\right\},\left\{0.5\right\}\right\}$	$\left\{\left\{0.4\right\},\left\{0\right\},\left\{0.4\right\}\right\}$	$\left\{\left\{0.2\right\},\left\{0\right\},\left\{0.6\right\}\right\}$
*A*_4_	$\left\{\left\{0.5\right\},\left\{0\right\},\left\{0.4\right\}\right\}$	$\left\{\left\{0.7\right\},\left\{0\right\},\left\{0.2\right\}\right\}$	$\left\{\left\{0.4\right\},\left\{0\right\},\left\{0.4\right\}\right\}$	$\left\{\left\{0.6\right\},\left\{0\right\},\left\{0.2\right\}\right\}$
*A*_5_	$\left\{\left\{0.6\right\},\left\{0\right\},\left\{0.3\right\}\right\}$	$\left\{\left\{0.7\right\},\left\{0\right\},\left\{0.2\right\}\right\}$	$\left\{\left\{0.4\right\},\left\{0\right\},\left\{0.2\right\}\right\}$	$\left\{\left\{0.7\right\},\left\{0\right\},\left\{0.2\right\}\right\}$

**Table 9:** The calculation results by different methods of Example 2.

Methods	$\mathrm{Score}\;\mathrm{Function}\;s\left(d_{i}\right)\;\left(i=1,2,3,4\right)$	Ranking Orders
Wei’s [38] method based on PFWA operator	$s\left(d_{1}\right)=-0.008,$ $s\left(d_{2}\right)=0.2718,$ $s\left(d_{3}\right)=0.1751,$ $s\left(d_{4}\right)=0.2302,$ $s\left(d_{5}\right)=0.4069$	$A_{5}≻A_{2}≻A_{4}≻A_{3}≻A_{1}$
Wang et al.’s [39] method based on PFWMM operator $\left(P=\left[1,1,1,1\right]\right)$	$s\left(d_{1}\right)=-0.2641,$ $s\left(d_{2}\right)=0.1238,$ $s\left(d_{3}\right)=0.0698,$ $s\left(d_{4}\right)=-0.1206,$ $s\left(d_{5}\right)=0.2071$	$A_{5}≻A_{2}≻A_{3}≻A_{4}≻A_{1}$
Wang and Liu’ [33] method based on IFSSWMSM operator $\left(k=2,\gamma =-2\right)$	$s\left(d_{1}\right)=-0.6369,$ $s\left(d_{2}\right)=-0.1851,$ $s\left(d_{3}\right)=-0.3354,$ $s\left(d_{4}\right)=-0.4193,$ $s\left(d_{5}\right)=-0.0462$	$A_{5}≻A_{2}≻A_{3}≻A_{4}≻A_{1}$
$\mathrm{Our}\;\mathrm{method}\;\mathrm{with}\;\mathrm{HPFSSWMSM}\;\mathrm{operator}\;\left(k=1,\gamma =-2\right)$	$s\left(d_{1}\right)=-0.6467,$ $s\left(d_{2}\right)=-0.2479,$ $s\left(d_{3}\right)=-0.3787,$ $s\left(d_{4}\right)=-0.4545,$ $s\left(d_{5}\right)=-0.1241$	$A_{5}≻A_{2}≻A_{3}≻A_{4}≻A_{1}$
$\mathrm{Our}\;\mathrm{method}\;\mathrm{with}\;\mathrm{HPFSSWMSM}\;\mathrm{operator}\;\left(k=1,\gamma \to 0\right)$	$s\left(d_{1}\right)=-0.9351,$ $s\left(d_{2}\right)=-0.8343,$ $s\left(d_{3}\right)=-0.8854,$ $s\left(d_{4}\right)=-0.8819,$ $s\left(d_{5}\right)=-0.8064$	$A_{5}≻A_{2}≻A_{4}≻A_{3}≻A_{1}$
$\mathrm{Our}\;\mathrm{method}\;\mathrm{with}\;\mathrm{HPFSSWMSM}\;\mathrm{operator}\;\left(k=2,\gamma =-2\right)$	$s\left(d_{1}\right)=-0.6369,$ $s\left(d_{2}\right)=-0.1851,$ $s\left(d_{3}\right)=-0.3354,$ $s\left(d_{4}\right)=-0.4193,$ $s\left(d_{5}\right)=-0.0462$	$A_{5}≻A_{2}≻A_{3}≻A_{4}≻A_{1}$
$\mathrm{Our}\;\mathrm{method}\;\mathrm{with}\;\mathrm{HPFSSWMSM}\;\mathrm{operator}\;\left(k=3,\gamma =-2\right)$	$s\left(d_{1}\right)=-0.4121,$ $s\left(d_{2}\right)=0.0912,$ $s\left(d_{3}\right)=-0.0287,$ $s\left(d_{4}\right)=-0.1440,$ $s\left(d_{5}\right)=0.2499$	$A_{5}≻A_{2}≻A_{3}≻A_{4}≻A_{1}$
$\mathrm{Our}\;\mathrm{method}\;\mathrm{with}\;\mathrm{HPFSSWMSM}\;\mathrm{operator}\;\left(k=4,\gamma =-2\right)$	$s\left(d_{1}\right)=0.2994,$ $s\left(d_{2}\right)=0.6295,$ $s\left(d_{3}\right)=0.5988,$ $s\left(d_{4}\right)=0.4982,$ $s\left(d_{5}\right)=0.7270$	$A_{5}≻A_{2}≻A_{3}≻A_{4}≻A_{1}$

**Table 10:** The adjusted evaluation values of Table 8 given by DM *D*_2_.

	*G*_1_	*G*_2_	*G*_3_	*G*_4_
*A*_1_	$\left\{\left\{0.4\right\},\left\{0.1\right\},\left\{0.5\right\}\right\}$	$\left\{\left\{0.6\right\},\left\{0.02\right\},\left\{0.2\right\}\right\}$	$\left\{\left\{0.5\right\},\left\{0.06\right\},\left\{0.4\right\}\right\}$	$\left\{\left\{0.5\right\},\left\{0.14\right\},\left\{0.3\right\}\right\}$
*A*_2_	$\left\{\left\{0.5\right\},\left\{0.09\right\},\left\{0.4\right\}\right\}$	$\left\{\left\{0.6\right\},\left\{0\right\},\left\{0.2\right\}\right\}$	$\left\{\left\{0.6\right\},\left\{0.07\right\},\left\{0.3\right\}\right\}$	$\left\{\left\{0.7\right\},\left\{0\right\},\left\{0.3\right\}\right\}$
*A*_3_	$\left\{\left\{0.4\right\},\left\{0\right\},\left\{0.5\right\}\right\}$	$\left\{\left\{0.3\right\},\left\{0.1\right\},\left\{0.5\right\}\right\}$	$\left\{\left\{0.4\right\},\left\{0.02\right\},\left\{0.4\right\}\right\}$	$\left\{\left\{0.2\right\},\left\{0\right\},\left\{0.6\right\}\right\}$
*A*_4_	$\left\{\left\{0.5\right\},\left\{0.08\right\},\left\{0.4\right\}\right\}$	$\left\{\left\{0.7\right\},\left\{0.01\right\},\left\{0.2\right\}\right\}$	$\left\{\left\{0.4\right\},\left\{0\right\},\left\{0.4\right\}\right\}$	$\left\{\left\{0.6\right\},\left\{0.04\right\},\left\{0.2\right\}\right\}$
*A*_5_	$\left\{\left\{0.6\right\},\left\{0\right\},\left\{0.3\right\}\right\}$	$\left\{\left\{0.7\right\},\left\{0.05\right\},\left\{0.2\right\}\right\}$	$\left\{\left\{0.4\right\},\left\{0.2\right\},\left\{0.2\right\}\right\}$	$\left\{\left\{0.7\right\},\left\{0\right\},\left\{0.2\right\}\right\}$

**Table 11:** A comprehensive comparison of different methods.

	Capture Relationship among Attributes	Deal with Evaluation Information with Several Elements in MD and NMD	Based on a Flexible Operational Rule	Capture Neutral Membership Degree
Wang and Liu’s [33] method	Yes	No	No	No
Wei’s [38] method	No	No	No	Yes
Wang et al.’s [39] method	Yes	No	No	Yes
The proposed method	Yes	Yes	Yes	Yes

## Data Availability

The data presented in this study are available in article.

## Appendix A

**Proof** **of** **Theorem** **2.** According to the operations for HPFEs, we can obtain

$$\prod_{j=1}^{k}d_{i_{j}}=\cup_{\chi_{i_{j}}\in h_{i_{j}},\phi_{i_{j}}\in g_{i_{j}},\tau_{i_{j}}\in t_{i_{j}}}\left\{\left\{{\left(\sum_{j=1}^{k}\chi_{i_{j}}-1\right)}^{\frac{1}{\gamma }}\right\},\left\{1-{\left(\sum_{j=1}^{k}{\left(1-\phi_{i_{j}}\right)}^{\gamma }-1\right)}^{\frac{1}{\gamma }}\right\},\left\{1-{\left(\sum_{j=1}^{k}{\left(1-\tau_{i_{j}}\right)}^{\gamma }-1\right)}^{\frac{1}{\gamma }}\right\}\right\},$$

and,

$$\sum_{1\leq i_{1}<\ldots <i_{k}\leq n}\prod_{j=1}^{k}d_{i_{j}}=\cup_{\chi_{i_{j}}\in h_{i_{j}},\phi_{i_{j}}\in g_{i_{j}},\tau_{i_{j}}\in t_{i_{j}}}\left\{\left\{1-{\left(\sum_{1\leq i_{1}<\ldots <i_{k}\leq n}{\left(1-{\left(\sum_{j=1}^{k}\chi_{i_{j}}-1\right)}^{\frac{1}{\gamma }}\right)}^{\gamma }-1\right)}^{\frac{1}{\gamma }}\right\},\right.$$

$$\left.\left\{{\left(\sum_{1\leq i_{1}<\ldots <i_{k}\leq n}{\left(1-{\left(\sum_{j=1}^{k}{\left(1-\phi_{i_{j}}\right)}^{\gamma }-1\right)}^{\frac{1}{\gamma }}\right)}^{\gamma }-1\right)}^{\frac{1}{\gamma }}\right\},\left\{{\left(\sum_{1\leq i_{1}<\ldots <i_{k}\leq n}{\left(1-{\left(\sum_{j=1}^{k}{\left(1-\tau_{i_{j}}\right)}^{\gamma }-1\right)}^{\frac{1}{\gamma }}\right)}^{\gamma }-1\right)}^{\frac{1}{\gamma }}\right\}\right\}.$$

Further,

$$\frac{\sum_{1\leq i_{1}<\ldots <i_{k}\leq n}\prod_{j=1}^{k}d_{i_{j}}}{C_{n}^{k}}=\cup_{\chi_{i_{j}}\in h_{i_{j}},\phi_{i_{j}}\in g_{i_{j}},\tau_{i_{j}}\in t_{i_{j}}}\left\{\left\{1-{\left(\frac{1}{C_{n}^{k}}\sum_{1\leq i_{1}<\ldots <i_{k}\leq n}{\left(1-{\left(\sum_{j=1}^{k}\chi_{i_{j}}-1\right)}^{\frac{1}{\gamma }}\right)}^{\gamma }+1\right)}^{\frac{1}{\gamma }}\right\},\right.$$

$$\left\{{\left(\frac{1}{C_{n}^{k}}\sum_{1\leq i_{1}<\ldots <i_{k}\leq n}{\left(1-{\left(\sum_{j=1}^{k}{\left(1-\phi_{i_{j}}\right)}^{\gamma }-1\right)}^{\frac{1}{\gamma }}\right)}^{\gamma }+1\right)}^{\frac{1}{\gamma }}\right\},$$

$$\left.\left\{{\left(\frac{1}{C_{n}^{k}}\sum_{1\leq i_{1}<\ldots <i_{k}\leq n}{\left(1-{\left(\sum_{j=1}^{k}{\left(1-\tau_{i_{j}}\right)}^{\gamma }-1\right)}^{\frac{1}{\gamma }}\right)}^{\gamma }+1\right)}^{\frac{1}{\gamma }}\right\}\right\}.$$

Therefore,

$$HPFSSMSM^{\left(k\right)}\left(d_{1},d_{2},\ldots ,d_{n}\right)={\left(\frac{\sum_{1\leq i_{1}<\ldots <i_{k}\leq n}\prod_{j=1}^{k}d_{i_{j}}}{C_{n}^{k}}\right)}^{1/k}=$$

$$\cup_{\chi_{i_{j}}\in h_{i_{j}},\phi_{i_{j}}\in g_{i_{j}},\tau_{i_{j}}\in t_{i_{j}}}\left\{\left\{{\left(\frac{1}{k}{\left(1-{\left(\frac{1}{C_{n}^{k}}\sum_{1\leq i_{1}<\ldots <i_{k}\leq n}{\left(1-{\left(\sum_{j=1}^{k}\chi_{i_{j}}^{\gamma }-1\right)}^{\frac{1}{\gamma }}\right)}^{\gamma }+1\right)}^{\frac{1}{\gamma }}\right)}^{\gamma }-\left(\frac{1}{k}-1\right)\right)}^{\frac{1}{\gamma }}\right\},\right.$$

$$\left\{1-{\left(\frac{1}{k}{\left(1-{\left(\frac{1}{C_{n}^{k}}\sum_{1\leq i_{1}<\ldots <i_{k}\leq n}{\left(1-{\left(\sum_{j=1}^{k}{\left(1-\phi_{i_{j}}\right)}^{\gamma }-1\right)}^{\frac{1}{\gamma }}\right)}^{\gamma }+1\right)}^{\frac{1}{\gamma }}\right)}^{\gamma }-\left(\frac{1}{k}-1\right)\right)}^{\frac{1}{\gamma }}\right\},$$

$$\left.\left\{1-{\left(\frac{1}{k}{\left(1-{\left(\frac{1}{C_{n}^{k}}\sum_{1\leq i_{1}<\ldots <i_{k}\leq n}{\left(1-{\left(\sum_{j=1}^{k}{\left(1-\tau_{i_{j}}\right)}^{\gamma }-1\right)}^{\frac{1}{\gamma }}\right)}^{\gamma }+1\right)}^{\frac{1}{\gamma }}\right)}^{\gamma }-\left(\frac{1}{k}-1\right)\right)}^{\frac{1}{\gamma }}\right\}\right\}$$

$$\mathrm{Let}\;\chi ={\left(\frac{1}{k}{\left(1-{\left(\frac{1}{C_{n}^{k}}\sum_{1\leq i_{1}<\ldots <i_{k}\leq n}{\left(1-{\left(\sum_{j=1}^{k}\chi_{i_{j}}^{\gamma }-1\right)}^{\frac{1}{\gamma }}\right)}^{\gamma }+1\right)}^{\frac{1}{\gamma }}\right)}^{\gamma }-\left(\frac{1}{k}-1\right)\right)}^{\frac{1}{\gamma }},$$

$$\phi =1-{\left(\frac{1}{k}{\left(1-{\left(\frac{1}{C_{n}^{k}}\sum_{1\leq i_{1}<\ldots <i_{k}\leq n}{\left(1-{\left(\sum_{j=1}^{k}{\left(1-\phi_{i_{j}}\right)}^{\gamma }-1\right)}^{\frac{1}{\gamma }}\right)}^{\gamma }+1\right)}^{\frac{1}{\gamma }}\right)}^{\gamma }-\left(\frac{1}{k}-1\right)\right)}^{\frac{1}{\gamma }}$$

$$\tau =1-{\left(\frac{1}{k}{\left(1-{\left(\frac{1}{C_{n}^{k}}\sum_{1\leq i_{1}<\ldots <i_{k}\leq n}{\left(1-{\left(\sum_{j=1}^{k}{\left(1-\tau_{i_{j}}\right)}^{\gamma }-1\right)}^{\frac{1}{\gamma }}\right)}^{\gamma }+1\right)}^{\frac{1}{\gamma }}\right)}^{\gamma }-\left(\frac{1}{k}-1\right)\right)}^{\frac{1}{\gamma }}.$$

Then, it is easy to prove that $\chi ,\phi ,\tau ,\in \left[0,1\right]$.

Since $\chi_{i_{j}}+\phi_{i_{j}}+\tau_{i_{j}}\leq 1$, then, we can obtain

$$\chi +\phi +\tau ={\left(\frac{1}{k}{\left(1-{\left(\frac{1}{C_{n}^{k}}\sum_{1\leq i_{1}<\ldots <i_{k}\leq n}{\left(1-{\left(\sum_{j=1}^{k}\chi_{i_{j}}^{\gamma }-1\right)}^{\frac{1}{\gamma }}\right)}^{\gamma }+1\right)}^{\frac{1}{\gamma }}\right)}^{\gamma }-\left(\frac{1}{k}-1\right)\right)}^{\frac{1}{\gamma }}$$

$$+1-{\left(\frac{1}{k}{\left(1-{\left(\frac{1}{C_{n}^{k}}\sum_{1\leq i_{1}<\ldots <i_{k}\leq n}{\left(1-{\left(\sum_{j=1}^{k}{\left(1-\phi_{i_{j}}\right)}^{\gamma }-1\right)}^{\frac{1}{\gamma }}\right)}^{\gamma }+1\right)}^{\frac{1}{\gamma }}\right)}^{\gamma }-\left(\frac{1}{k}-1\right)\right)}^{\frac{1}{\gamma }}$$

$$+1-{\left(\frac{1}{k}{\left(1-{\left(\frac{1}{C_{n}^{k}}\sum_{1\leq i_{1}<\ldots <i_{k}\leq n}{\left(1-{\left(\sum_{j=1}^{k}{\left(1-\tau_{i_{j}}\right)}^{\gamma }-1\right)}^{\frac{1}{\gamma }}\right)}^{\gamma }+1\right)}^{\frac{1}{\gamma }}\right)}^{\gamma }-\left(\frac{1}{k}-1\right)\right)}^{\frac{1}{\gamma }}$$

$$\leq {\left(\frac{1}{k}{\left(1-{\left(\frac{1}{C_{n}^{k}}\sum_{1\leq i_{1}<\ldots <i_{k}\leq n}{\left(1-{\left(\sum_{j=1}^{k}\chi_{i_{j}}-1\right)}^{\frac{1}{\gamma }}\right)}^{\gamma }+1\right)}^{\frac{1}{\gamma }}\right)}^{\gamma }-\left(\frac{1}{k}-1\right)\right)}^{\frac{1}{\gamma }}$$

$$+1-{\left(\frac{1}{k}{\left(1-{\left(\frac{1}{C_{n}^{k}}\sum_{1\leq i_{1}<\ldots <i_{k}\leq n}{\left(1-{\left(\sum_{j=1}^{k}{\left(\chi_{i_{j}}+\tau_{i_{j}}\right)}^{\gamma }-1\right)}^{\frac{1}{\gamma }}\right)}^{\gamma }+1\right)}^{\frac{1}{\gamma }}\right)}^{\gamma }-\left(\frac{1}{k}-1\right)\right)}^{\frac{1}{\gamma }}$$

$$+1-{\left(\frac{1}{k}{\left(1-{\left(\frac{1}{C_{n}^{k}}\sum_{1\leq i_{1}<\ldots <i_{k}\leq n}{\left(1-{\left(\sum_{j=1}^{k}{\left(1-\tau_{i_{j}}\right)}^{\gamma }-1\right)}^{\frac{1}{\gamma }}\right)}^{\gamma }+1\right)}^{\frac{1}{\gamma }}\right)}^{\gamma }-\left(\frac{1}{k}-1\right)\right)}^{\frac{1}{\gamma }}$$

$$\leq {\left(\frac{1}{k}{\left(1-{\left(\frac{1}{C_{n}^{k}}\sum_{1\leq i_{1}<\ldots <i_{k}\leq n}{\left(1-{\left(\sum_{j=1}^{k}\chi_{i_{j}}-1\right)}^{\frac{1}{\gamma }}\right)}^{\gamma }+1\right)}^{\frac{1}{\gamma }}\right)}^{\gamma }-\left(\frac{1}{k}-1\right)\right)}^{\frac{1}{\gamma }}$$

$$+1-{\left(\frac{1}{k}{\left(1-{\left(\frac{1}{C_{n}^{k}}\sum_{1\leq i_{1}<\ldots <i_{k}\leq n}{\left(1-{\left(\sum_{j=1}^{k}\chi_{i_{j}}+\sum_{j=1}^{k}\tau_{i_{j}}-1\right)}^{\frac{1}{\gamma }}\right)}^{\gamma }+1\right)}^{\frac{1}{\gamma }}\right)}^{\gamma }-\left(\frac{1}{k}-1\right)\right)}^{\frac{1}{\gamma }}$$

$$+1-{\left(\frac{1}{k}{\left(1-{\left(\frac{1}{C_{n}^{k}}\sum_{1\leq i_{1}<\ldots <i_{k}\leq n}{\left(1-{\left(\sum_{j=1}^{k}{\left(1-\tau_{i_{j}}\right)}^{\gamma }-1\right)}^{\frac{1}{\gamma }}\right)}^{\gamma }+1\right)}^{\frac{1}{\gamma }}\right)}^{\gamma }-\left(\frac{1}{k}-1\right)\right)}^{\frac{1}{\gamma }}$$

≤−1+2=1.

Which means the aggregated value by the HPFSSMSM operator is also an HPFE. Therefore, the proof of Theorem 2 is complete. □

**Proof** **of** **Theorem** **3.** Since $d=\left(h,g,t\right)$, based on Theorem 2, we obtain

$$HPFSSMSM^{\left(k\right)}\left(d_{1},d_{2},\ldots ,d_{n}\right)={\left(\frac{\sum_{1\leq i_{1}<\ldots <i_{k}\leq n}\prod_{j=1}^{k}d}{C_{n}^{k}}\right)}^{1/k}={\left(\frac{\sum_{1\leq i_{1}<\ldots <i_{k}\leq n}d^{k}}{C_{n}^{k}}\right)}^{1/k}={\left(\frac{C_{n}^{k}d^{k}}{C_{n}^{k}}\right)}^{1/k}={\left(d^{k}\right)}^{\frac{1}{k}}=d.$$

Thus, the proof of Theorem 3 is completed. □

**Proof** **of** **Theorem** **4.** Since $d_{j}\geq d_{j}^{\prime }$ holds for all j=1,2,…,n, we can gather that

$$\prod_{j=1}^{k}d_{i_{j}}\geq \prod_{j=1}^{k}d_{i_{j}}^{\prime }\;\mathrm{and}\;\sum_{1\leq i_{1}<\ldots <i_{k}\leq n}\prod_{j=1}^{k}d_{i_{j}}\leq \sum_{1\leq i_{1}<\ldots <i_{k}\leq n}\prod_{j=1}^{k}d_{i_{j}}^{\prime }.$$

Therefore,

$$\frac{\sum_{1\leq i_{1}<\ldots <i_{k}\leq n}\prod_{j=1}^{k}d_{i_{j}}}{C_{n}^{k}}\geq \frac{\sum_{1\leq i_{1}<\ldots <i_{k}\leq n}\prod_{j=1}^{k}d_{i_{j}}^{\prime }}{C_{n}^{k}}.$$

Thus,

$${\left(\frac{\sum_{1\leq i_{1}<\ldots <i_{k}\leq n}\prod_{j=1}^{k}d_{i_{j}}}{C_{n}^{k}}\right)}^{1/k}\geq {\left(\frac{\sum_{1\leq i_{1}<\ldots <i_{k}\leq n}\prod_{j=1}^{k}d_{i_{j}}^{\prime }}{C_{n}^{k}}\right)}^{1/k},$$

i.e.,

$$HPFSSMSM^{\left(k\right)}\left(d_{1},d_{2},\ldots ,d_{n}\right)\geq HPFSSMSM^{\left(k\right)}\left(d_{1}^{\prime },d_{2}^{\prime },\ldots ,d_{n}^{\prime }\right).$$

Therefore, the proof of Theorem 4 is complete. □

**Proof** **of** **Theorem** **5.** According to Theorem 3 and Theorem 4, we can obtain

$$HPFSSMSM^{\left(k\right)}\left(d_{1},d_{2},\ldots ,d_{n}\right)\geq HPFSSMSM^{\left(k\right)}\left(d^{-},d^{-},\ldots ,d^{-}\right)=d^{-},$$

and

$$HPFSSMSM^{\left(k\right)}\left(d_{1},d_{2},\ldots ,d_{n}\right)\leq HPFSSMSM^{\left(k\right)}\left(d^{+},d^{+},\ldots ,d^{+}\right)=d^{+}.$$

Thus, we can obtain

$$d^{-}\leq HPFSSMSM^{\left(k\right)}\left(d_{1},d_{2},\ldots ,d_{n}\right)\leq d^{+}.$$

Therefore, the proof of Theorem 5 is complete. □

**Proof** **of** **Theorem** **6.**

$$\mathrm{Let}\;f{\left(k\right)}_{\chi }={\left(\frac{1}{k}{\left(1-{\left(\frac{1}{C_{n}^{k}}\sum_{1\leq i_{1}<\ldots <i_{k}\leq n}{\left(1-{\left(\sum_{j=1}^{k}\chi_{i_{j}}-1\right)}^{\frac{1}{\gamma }}\right)}^{\gamma }+1\right)}^{\frac{1}{\gamma }}\right)}^{\gamma }-\left(\frac{1}{k}-1\right)\right)}^{\frac{1}{\gamma }},$$

$$f{\left(k\right)}_{\phi }=1-{\left(\frac{1}{k}{\left(1-{\left(\frac{1}{C_{n}^{k}}\sum_{1\leq i_{1}<\ldots <i_{k}\leq n}{\left(1-{\left(\sum_{j=1}^{k}{\left(1-\phi_{i_{j}}\right)}^{\gamma }-1\right)}^{\frac{1}{\gamma }}\right)}^{\gamma }+1\right)}^{\frac{1}{\gamma }}\right)}^{\gamma }-\left(\frac{1}{k}-1\right)\right)}^{\frac{1}{\gamma }},$$

$$f{\left(k\right)}_{\tau }=1-{\left(\frac{1}{k}{\left(1-{\left(\frac{1}{C_{n}^{k}}\sum_{1\leq i_{1}<\ldots <i_{k}\leq n}{\left(1-{\left(\sum_{j=1}^{k}{\left(1-\tau_{i_{j}}\right)}^{\gamma }-1\right)}^{\frac{1}{\gamma }}\right)}^{\gamma }+1\right)}^{\frac{1}{\gamma }}\right)}^{\gamma }-\left(\frac{1}{k}-1\right)\right)}^{\frac{1}{\gamma }}.$$

Based on Theorem 1, we have $HPFSSMSM^{\left(k\right)}\left(d_{1},d_{2},\ldots ,d_{n}\right)=\left(f{\left(k\right)}_{\chi },f{\left(k\right)}_{\phi },f{\left(k\right)}_{\tau }\right)$. Based on the Lemma 1, we can obtain

$$f{\left(k\right)}_{\chi }={\left(\frac{1}{k}{\left(1-{\left(\frac{1}{C_{n}^{k}}\sum_{1\leq i_{1}<\ldots <i_{k}\leq n}{\left(1-{\left(\sum_{j=1}^{k}\chi_{i_{j}}-1\right)}^{\frac{1}{\gamma }}\right)}^{\gamma }+1\right)}^{\frac{1}{\gamma }}\right)}^{\gamma }-\left(\frac{1}{k}-1\right)\right)}^{\frac{1}{\gamma }}$$

$$={\left(\frac{1}{k}{\left(1-{\left(\frac{1}{C_{n}^{k}}\sum_{1\leq i_{1}<\ldots <i_{k}\leq n}{\left(1-{\left(\sum_{j=1}^{k}\chi_{i_{j}}-1\right)}^{\frac{1}{\gamma }}\right)}^{\gamma }+1\right)}^{\frac{1}{\gamma }}\right)}^{\gamma }-\frac{1}{k}+1\right)}^{\frac{1}{\gamma }}$$

$$={\left(\frac{1}{k}\left({\left(1-{\left(\prod_{1\leq i_{1}<\ldots <i_{k}\leq n}{\left(1-{\left(\sum_{j=1}^{k}\chi_{i_{j}}-1\right)}^{\frac{1}{\gamma }}\right)}^{\frac{\gamma }{C_{n}^{k}}}+1\right)}^{\frac{1}{\gamma }}\right)}^{\gamma }-1\right)+1\right)}^{\frac{1}{\gamma }}$$

$$\leq {\left(\left({\left(1-{\left(\prod_{1\leq i_{1}<\ldots <i_{k}\leq n}{\left(1-{\left(\sum_{j=1}^{k}\chi_{i_{j}}-1\right)}^{\frac{1}{\gamma }}\right)}^{\frac{\gamma }{C_{n}^{k}}}+1\right)}^{\frac{1}{\gamma }}\right)}^{\gamma }-1\right)+1\right)}^{\frac{1}{\gamma }}$$

$$=1-{\left(\prod_{1\leq i_{1}<\ldots <i_{k}\leq n}{\left(1-{\left(\sum_{j=1}^{k}\chi_{i_{j}}-1\right)}^{\frac{1}{\gamma }}\right)}^{\frac{\gamma }{C_{n}^{k}}}+1\right)}^{\frac{1}{\gamma }}$$

$$\leq 1-{\left(\prod_{1\leq i_{1}<\ldots <i_{k}\leq n}{\left(1-{\left(\sum_{j=1}^{k}\chi_{i_{j}}-1\right)}^{\frac{1}{\gamma }}\right)}^{\frac{\gamma }{C_{n}^{k}}}\right)}^{\frac{1}{\gamma }}$$

$$=1-\prod_{1\leq i_{1}<\ldots <i_{k}\leq n}{\left(1-{\left(\sum_{j=1}^{k}\chi_{i_{j}}-1\right)}^{\frac{1}{\gamma }}\right)}^{\frac{1}{C_{n}^{k}}}$$

Then, we can take the following proof by using contradiction method. We suppose that the function $f{\left(k\right)}_{\chi }$ monotonically increases with the increase in *k*, and then, it follows that

$$f{\left(n\right)}_{\chi }>f{\left(n-1\right)}_{\chi }>\ldots >f{\left(1\right)}_{\chi }$$

Since,

$$f{\left(1\right)}_{\chi }\leq 1-\prod_{i=1}^{n}{\left(1-{\left(\sum_{j=1}^{1}\chi_{i_{j}}-1\right)}^{\frac{1}{\gamma }}\right)}^{\frac{1}{C_{n}^{1}}}=1-\prod_{i=1}^{n}{\left(1-{\left(\chi_{i}-1\right)}^{\frac{1}{\gamma }}\right)}^{\frac{1}{n}},$$

then $f{\left(n\right)}_{\chi }>f{\left(1\right)}_{\chi }$, and

$$f{\left(n\right)}_{\chi }=1-\prod_{1\leq i_{1}<\ldots <i_{k}\leq n}{\left(1-{\left(\sum_{j=1}^{k}\chi_{i_{j}}-1\right)}^{\frac{1}{\gamma }}\right)}^{\frac{1}{C_{n}^{k}}}=1-\prod_{1\leq i_{1}<\ldots <i_{k}\leq n}\left(1-{\left(\sum_{j=1}^{n}\chi_{i_{j}}-1\right)}^{\frac{1}{\gamma }}\right)=1-\left(1-{\left(\sum_{j=1}^{n}\chi_{i_{j}}-1\right)}^{\frac{1}{\gamma }}\right)$$

(A1) $$=1-\left(1-{\left(\sum_{i=1}^{n}\chi_{i}-1\right)}^{\frac{1}{\gamma }}\right)={\left(\sum_{i=1}^{n}\chi_{i}-1\right)}^{\frac{1}{\gamma }}>1-\prod_{i=1}^{n}{\left(1-{\left(\chi_{i}-1\right)}^{\frac{1}{\gamma }}\right)}^{\frac{1}{n}}=f{\left(1\right)}_{\chi }$$

However, according to the Lemma 1, we have

(A2) $$1-\prod_{1\leq i_{1}\leq n}{\left(1-{\left(\chi_{i_{1}}-1\right)}^{\frac{1}{\gamma }}\right)}^{\frac{1}{n}}\geq 1-\frac{1}{n}\sum_{i=1}^{n}\left(1-{\left(\chi_{i}-1\right)}^{\frac{1}{\gamma }}\right)=1-\sum_{i=1}^{n}{\left(\chi_{i}-1\right)}^{\frac{1}{\gamma }}>{\left(\sum_{i=1}^{n}\chi_{i}-1\right)}^{\frac{1}{\gamma }}.$$

Clearly, Equation (A1) is a contradiction to Equation (A2). Therefore, the function $f{\left(k\right)}_{\chi }$ monotonically decreases with the increase in *k*. Similarly, we can prove the function $f{\left(k\right)}_{\phi }$ and $f{\left(k\right)}_{\tau }$ monotonically increase with the increase in *k*.

Then, by utilizing the score function of HPFE, we have

(A3) $$S\left(HPFSSMSM^{\left(k\right)}\left(d_{1},d_{2},\ldots ,d_{n}\right)\right)=\frac{1}{\#h}\sum_{\chi \in h}f{\left(k\right)}_{\chi }-\frac{1}{\#t}\sum_{\tau \in t}f{\left(k\right)}_{\tau }.$$

For any k∈[0,n−1], we can obtain

$$S\left(HPFSSMSM^{\left(k+1\right)}\left(d_{1},d_{2},\ldots ,d_{n}\right)\right)-S\left(HPFSSMSM^{\left(k\right)}\left(d_{1},d_{2},\ldots ,d_{n}\right)\right)$$

$$=\frac{1}{\#h}\sum_{\chi \in h}f{\left(k+1\right)}_{\chi }-\frac{1}{\#t}\sum_{\tau \in t}f{\left(k+1\right)}_{\tau }-\frac{1}{\#h}\sum_{\chi \in h}f{\left(k\right)}_{\chi }+\frac{1}{\#t}\sum_{\tau \in t}f{\left(k\right)}_{\tau }$$

$$=\left(\frac{1}{\#h}\sum_{\chi \in h}f{\left(k+1\right)}_{\chi }-\frac{1}{\#h}\sum_{\chi \in h}f{\left(k\right)}_{\chi }\right)+\left(\frac{1}{\#t}\sum_{\tau \in t}f{\left(k\right)}_{\tau }-\frac{1}{\#t}\sum_{\tau \in t}f{\left(k+1\right)}_{\tau }\right)$$

$$=\frac{1}{\#h}\left(\sum_{\chi \in h}f{\left(k+1\right)}_{\chi }-\sum_{\chi \in h}f{\left(k\right)}_{\chi }\right)+\frac{1}{\#t}\left(\sum_{\tau \in t}f{\left(k\right)}_{\tau }-\sum_{\tau \in t}f{\left(k+1\right)}_{\tau }\right)$$

$$=\frac{1}{\#h}\sum_{\chi \in h}\left(f{\left(k+1\right)}_{\chi }-f{\left(k\right)}_{\chi }\right)+\frac{1}{\#t}\sum_{\chi \in h}\left(f{\left(k\right)}_{\tau }-f{\left(k+1\right)}_{\tau }\right)$$

Since the values of function $f{\left(k\right)}_{\chi }$, $f{\left(k\right)}_{\tau }$ are non-negative numbers, and function $f{\left(k\right)}_{\chi }$ decreases and the function $f{\left(k\right)}_{\tau }$ increases with the increase in *k*, therefore,

(A4) $$S\left(HPFSSMSM^{\left(k+1\right)}\left(d_{1},d_{2},\ldots ,d_{n}\right)\right)\leq S\left(HPFSSMSM^{\left(k\right)}\left(d_{1},d_{2},\ldots ,d_{n}\right)\right).$$

According to the comparison methods between two HPFEs in Definition 3, we can obtain $HPFSSMSM^{\left(k+1\right)}\left(d_{1},d_{2},\ldots ,d_{n}\right)\leq HPFSSMSM^{\left(k\right)}\left(d_{1},d_{2},\ldots ,d_{n}\right)$ for any integer k∈[0,n−1], which completes the proof of Theorem 6. □

**Proof** **of** **Theorem** **7.** According to the operations for HPFEs, we can obtain

$$w_{i_{j}}d_{i_{j}}=\cup_{\chi_{i_{j}}\in h_{i_{j}},\phi_{i_{j}}\in g_{i_{j}},\tau_{i_{j}}\in t_{i_{j}}}\left\{\left\{1-{\left(w_{i_{j}}{\left(1-\chi_{i_{j}}\right)}^{\gamma }-\left(w_{i_{j}}-1\right)\right)}^{\frac{1}{\gamma }}\right\},\right.$$

$$\left.\left\{{\left(w_{i_{j}}\phi_{i_{j}}^{\gamma }-\left(w_{i_{j}}-1\right)\right)}^{\frac{1}{\gamma }}\right\},\left\{{\left(w_{i_{j}}\tau_{i_{j}}^{\gamma }-\left(w_{i_{j}}-1\right)\right)}^{\frac{1}{\gamma }}\right\}\right\},$$

and,

$$\prod_{j=1}^{k}w_{i_{j}}d_{i_{j}}=\cup_{\chi_{i_{j}}\in h_{i_{j}},\phi_{i_{j}}\in g_{i_{j}},\tau_{i_{j}}\in t_{i_{j}}}\left\{\left\{{\left(\sum_{j=1}^{k}{\left(1-{\left(w_{i_{j}}{\left(1-\chi_{i_{j}}\right)}^{\gamma }-\left(w_{i_{j}}-1\right)\right)}^{\frac{1}{\gamma }}\right)}^{\gamma }-1\right)}^{\frac{1}{\gamma }}\right\},\right.$$

$$\left.\left\{1-{\left(\sum_{j=1}^{k}{\left(1-{\left(w_{i_{j}}\phi_{i_{j}}^{\gamma }-\left(w_{i_{j}}-1\right)\right)}^{\frac{1}{\gamma }}\right)}^{\gamma }-1\right)}^{\frac{1}{\gamma }}\right\},\left\{1-{\left(\sum_{j=1}^{k}{\left(1-{\left(w_{i_{j}}\tau_{i_{j}}^{\gamma }-\left(w_{i_{j}}-1\right)\right)}^{\frac{1}{\gamma }}\right)}^{\gamma }-1\right)}^{\frac{1}{\gamma }}\right\}\right\}.$$

Further,

$$\sum_{1\leq i_{1}<\ldots <i_{k}\leq n}\prod_{j=1}^{k}w_{i_{j}}d_{i_{j}}=\cup_{\chi_{i_{j}}\in h_{i_{j}},\phi_{i_{j}}\in g_{i_{j}},\tau_{i_{j}}\in t_{i_{j}}}$$

$$\left\{\left\{1-{\left(\sum_{1\leq i_{1}<\ldots <i_{k}\leq n}{\left(1-{\left(\sum_{j=1}^{k}{\left(1-{\left(w_{i_{j}}{\left(1-\chi_{i_{j}}\right)}^{\gamma }-\left(w_{i_{j}}-1\right)\right)}^{\frac{1}{\gamma }}\right)}^{\gamma }-1\right)}^{\frac{1}{\gamma }}\right)}^{\gamma }-1\right)}^{\frac{1}{\gamma }}\right\},\right.$$

$$\left\{{\left(\sum_{1\leq i_{1}<\ldots <i_{k}\leq n}{\left(1-{\left(\sum_{j=1}^{k}{\left(1-{\left(w_{i_{j}}\phi_{i_{j}}^{\gamma }-\left(w_{i_{j}}-1\right)\right)}^{\frac{1}{\gamma }}\right)}^{\gamma }-1\right)}^{\frac{1}{\gamma }}\right)}^{\gamma }-1\right)}^{\frac{1}{\gamma }}\right\},$$

$$\left.\left\{{\left(\sum_{1\leq i_{1}<\ldots <i_{k}\leq n}{\left(1-{\left(\sum_{j=1}^{k}{\left(1-{\left(w_{i_{j}}\tau_{i_{j}}^{\gamma }-\left(w_{i_{j}}-1\right)\right)}^{\frac{1}{\gamma }}\right)}^{\gamma }-1\right)}^{\frac{1}{\gamma }}\right)}^{\gamma }-1\right)}^{\frac{1}{\gamma }}\right\}\right\},$$

and,

$$\frac{\sum_{1\leq i_{1}<\ldots <i_{k}\leq n}\prod_{j=1}^{k}w_{i_{j}}d_{i_{j}}}{C_{n}^{k}}=\cup_{\chi_{i_{j}}\in h_{i_{j}},\phi_{i_{j}}\in g_{i_{j}},\tau_{i_{j}}\in t_{i_{j}}}$$

$$\left\{\left\{1-{\left(\frac{1}{C_{n}^{k}}\sum_{1\leq i_{1}<\ldots <i_{k}\leq n}{\left(1-{\left(\sum_{j=1}^{k}{\left(1-{\left(w_{i_{j}}{\left(1-\chi_{i_{j}}\right)}^{\gamma }-\left(w_{i_{j}}-1\right)\right)}^{\frac{1}{\gamma }}\right)}^{\gamma }-1\right)}^{\frac{1}{\gamma }}\right)}^{\gamma }+1\right)}^{\frac{1}{\gamma }}\right\},\right.$$

$$\left\{{\left(\frac{1}{C_{n}^{k}}\sum_{1\leq i_{1}<\ldots <i_{k}\leq n}{\left(1-{\left(\sum_{j=1}^{k}{\left(1-{\left(w_{i_{j}}\phi_{i_{j}}^{\gamma }-\left(w_{i_{j}}-1\right)\right)}^{\frac{1}{\gamma }}\right)}^{\gamma }-1\right)}^{\frac{1}{\gamma }}\right)}^{\gamma }+1\right)}^{\frac{1}{\gamma }}\right\},$$

$$\left.\left\{{\left(\frac{1}{C_{n}^{k}}\sum_{1\leq i_{1}<\ldots <i_{k}\leq n}{\left(1-{\left(\sum_{j=1}^{k}{\left(1-{\left(w_{i_{j}}\tau_{i_{j}}^{\gamma }-\left(w_{i_{j}}-1\right)\right)}^{\frac{1}{\gamma }}\right)}^{\gamma }-1\right)}^{\frac{1}{\gamma }}\right)}^{\gamma }+1\right)}^{\frac{1}{\gamma }}\right\}\right\}$$

Therefore,

$${\left(\frac{\sum_{1\leq i_{1}<\ldots <i_{k}\leq n}\prod_{j=1}^{k}w_{i_{j}}d_{i_{j}}}{C_{n}^{k}}\right)}^{1/k}=\cup_{\chi_{i_{j}}\in h_{i_{j}},\phi_{i_{j}}\in g_{i_{j}},\tau_{i_{j}}\in t_{i_{j}}}$$

$$\left\{\left\{{\left(\frac{1}{k}{\left(1-{\left(\frac{1}{C_{n}^{k}}\sum_{1\leq i_{1}<\ldots <i_{k}\leq n}{\left(1-{\left(\sum_{j=1}^{k}{\left(1-{\left(w_{i_{j}}{\left(1-\chi_{i_{j}}\right)}^{\gamma }-\left(w_{i_{j}}-1\right)\right)}^{\frac{1}{\gamma }}\right)}^{\gamma }-1\right)}^{\frac{1}{\gamma }}\right)}^{\gamma }+1\right)}^{\frac{1}{\gamma }}\right)}^{\gamma }-\left(\frac{1}{k}-1\right)\right)}^{\frac{1}{\gamma }}\right\},\right.$$

$$\left\{1-{\left(\frac{1}{k}{\left(1-{\left(\frac{1}{C_{n}^{k}}\sum_{1\leq i_{1}<\ldots <i_{k}\leq n}{\left(1-{\left(\sum_{j=1}^{k}{\left(1-{\left(w_{i_{j}}\phi_{i_{j}}^{\gamma }-\left(w_{i_{j}}-1\right)\right)}^{\frac{1}{\gamma }}\right)}^{\gamma }-1\right)}^{\frac{1}{\gamma }}\right)}^{\gamma }+1\right)}^{\frac{1}{\gamma }}\right)}^{\gamma }-\left(\frac{1}{k}-1\right)\right)}^{\frac{1}{\gamma }}\right\}$$

$$\left.\left\{1-{\left(\frac{1}{k}{\left(1-{\left(\frac{1}{C_{n}^{k}}\sum_{1\leq i_{1}<\ldots <i_{k}\leq n}{\left(1-{\left(\sum_{j=1}^{k}{\left(1-{\left(w_{i_{j}}\tau_{i_{j}}^{\gamma }-\left(w_{i_{j}}-1\right)\right)}^{\frac{1}{\gamma }}\right)}^{\gamma }-1\right)}^{\frac{1}{\gamma }}\right)}^{\gamma }+1\right)}^{\frac{1}{\gamma }}\right)}^{\gamma }-\left(\frac{1}{k}-1\right)\right)}^{\frac{1}{\gamma }}\right\}\right\}$$

Therefore, the proof of Theorem 7 is completed. □

**[Comprehensive matrix of subsection 5.1]**

$$d_{11}^{\prime }=\left\{\left\{0.0884,0.1109\right\},\left\{0.0181\right\},\left\{0.5498,0.6442,0.5640,0.6638,0.5695,0.6715,0.5848,0.6929\right\}\right\}\;d_{12}^{\prime }=\left\{\left\{0.0854\right\},\left\{0.2273,0.2378\right\},\left\{0.4204,0.4889,0.4484,0.5271\right\}\right\};\;d_{13}^{\prime }=\left\{\left\{0.1315,0.1676,0.1660,0.1993\right\},\right.\;\;\left.\left\{0.0286,0.0290,0.0501,0.0513,0.0295,0.0299,0.0528,0.0541\right\},\left\{0.5909\right\}\right\}\;\;d_{14}^{\prime }=\left\{\left\{0.0733,0.0820,0.0796,0.0883\right\},\left\{0.0587\right\},\right.\;\left.\left\{0.7012,0.7119,0.7311,0.7427,0.7371,0.7489,0.7702,0.7830\right\}\right\}\;\;d_{21}^{\prime }=\left\{\left\{0.2518,0.2667\right\},\left\{0.0706\right\},\left\{0.6154,0.6239\right\}\right\};\;d_{22}^{\prime }=\left\{\left\{0.2862,0.2949,0.3130,0.3210,0.3324,0.3400,0.3558,0.3629\right\},\left\{0.0312\right\},\left\{0.3429,0.3587\right\}\right\}\;\;d_{23}^{\prime }=\left\{\left\{0.2193,0.2439,0.2348,0.2584,0.2629,0.2848,0.2767,0.2979\right\},\left\{0.0606\right\},\left\{0.3982,0.4071\right\}\right\};\;d_{24}^{\prime }=\left\{\left\{0.1398\right\},\left\{0.0364\right\},\left\{0.7156,0.7266,0.7634,0.7768\right\}\right\}\;\;d_{31}^{\prime }=\left\{\left\{0.1651\right\},\left\{0.0121,0.0222\right\},\left\{0.7284,0.7482\right\}\right\};\;d_{32}^{\prime }=\left\{\left\{0.4031,0.4223,0.4075,0.4264\right\},\left\{0.0367\right\},\left\{0.4558,0.4632\right\}\right\};\;d_{33}^{\prime }=\left\{\left\{0.1686,0.1932,0.1755,0.1998,0.1843,0.2081,0.1910,0.2144\right\},\left\{0.0692,0.0900\right\},\left\{0.6699\right\}\right\}\;\;d_{34}^{\prime }=\left\{\left\{0.2142,0.2331,0.2170,0.2357\right\},\left\{0.0824,0.0884\right\},\left\{0.6037\right\}\right\}\;\;d_{41}^{\prime }=\left\{\left\{0.0653,0.0741,0.0741,0.0827\right\},\left\{0.0550\right\},\left\{0.6949,0.7068\right\}\right\};\;d_{42}^{\prime }=\left\{\left\{0.3421,0.3449\right\},\left\{0.0414\right\},\left\{0.5404\right\}\right\};\;d_{43}^{\prime }=\left\{\left\{0.1458\right\},\left\{0.0357\right\},\left\{0.6616,0.7337\right\}\right\};\;d_{44}^{\prime }=\left\{\left\{0.0810,0.1018,0.0891,0.1096,0.1034,0.1232,0.1111,0.1306\right\},\left\{0.0227\right\},\left\{0.6837,0.7178\right\}\right\}□$$

**[Comprehensive matrix of subsection 5.2]**

$$d_{11}^{\prime }=\left\{\left\{0.0833,0.0909\right\},\left\{0.1831\right\},\left\{0.6674,0.6989,0.7435,0.7627,0.6795,0.7089,0.7508,0.7690\right\}\right\}$$

$$d_{12}^{\prime }=\left\{\left\{0.0562\right\},\left\{0.2857,0.2931\right\},\left\{0.5385,0.6388,0.5459,0.6434\right\}\right\};$$

$$d_{13}^{\prime }=\left\{\left\{0.0646,0.0694,0.0653,0.0702\right\},\right.\;$$

$$\left.\left\{0.1132,0.1306,0.1156,0.1330,0.1345,0.1511,0.1368,0.1533\right\},\left\{0.6471\right\}\right\};$$

$$d_{14}^{\prime }=\left\{\left\{0.0691,0.0818,0.0719,0.0857\right\},\left\{0.0775\right\},\right.$$

$$\left.\left\{0.7036,0.7554,0.7470,0.7857,0.7123,0.7613,0.7533,0.7903\right\}\right\};$$

$$d_{21}^{\prime }=\left\{\left\{0.0691,0.0818,0.0719,0.0857\right\},\left\{0.0775\right\},\right.$$

$$\left.\left\{0.7063,0.7554,0.7470,0.7857,0.7123,0.7613,0.7533,0.7903\right\}\right\};$$

$$d_{22}^{\prime }=\left\{\left\{0.2273,0.2604\right\},\left\{0.0931\right\},\left\{0.6170,0.6305\right\}\right\};$$

$$d_{23}^{\prime }=\left\{\left\{0.1052,0.1071,0.1059,0.1079,0.2916,0.3072,0.2874,0.3137\right\},\left\{0.0706\right\},\left\{0.4362,0.4955\right\}\right\}\;$$

$$d_{24}^{\prime }=\left\{\left\{0.1935,0.2143,0.1981,0.2198,0.2446,0.2787,0.2519,0.2881\right\},\left\{0.1187\right\},\left\{0.5164,0.5262\right\}\right\}\;$$

$$d_{31}^{\prime }=\left\{\left\{0.1935,0.2143,0.1981,0.2198,0.2446,0.2787,0.2519,0.2881\right\},\left\{0.1187\right\},\left\{0.5164,0.5262\right\}\right\};$$

$$d_{32}^{\prime }=\left\{\left\{0.1152\right\},\left\{0.0370\right\},\left\{0.7457,0.7671,0.7704,0.7880,\right\}\right\};$$

$$d_{33}^{\prime }=\left\{\left\{0.0546\right\},\left\{0.248,0.0396\right\},\left\{0.7505,0.8238\right\}\right\};$$

$$d_{34}^{\prime }=\left\{\left\{0.3329,0.3474,0.3569,0.3736\right\},\left\{0.0582\right\},\left\{0.4747,0.5024\right\}\right\};$$

$$d_{41}^{\prime }=\left\{\left\{0.3329,0.3474,0.3569,0.3736\right\},\left\{0.0582\right\},\left\{0.4747,0.5024\right\}\right\};$$

$$d_{42}^{\prime }=\left\{\left\{0.1604,0.1851,0.1634,0.1891,0.1770,0.2076,0.1806,0.2127\right\},\left\{0.1190,0.1241\right\},\left\{0.6735\right\}\right\}\;$$

$$d_{43}^{\prime }=\left\{\left\{0.1115,0.1226,0.1430,0.1541\right\},\left\{0.0875,0.0957\right\},\left\{0.6570\right\}\right\};$$

$$d_{44}^{\prime }=\left\{\left\{0.0593,0.733,0.0618,0.0771\right\},\left\{0.1447\right\},\left\{0.7120,0.7369\right\}\right\}.$$
